# Sulfur-Phenolate Exchange as a Mild, Fast, and High-Yielding Method toward the Synthesis of Sulfonamides

**DOI:** 10.1021/acs.orglett.2c04292

**Published:** 2023-01-31

**Authors:** Alyssa F. J. van den Boom, Han Zuilhof

**Affiliations:** † Laboratory of Organic Chemistry, Wageningen University, Stippeneng 4, 6708 WE Wageningen, The Netherlands; ‡ School of Pharmaceutical Science and Technology, Tianjin University, 92 Weijin Road, Tianjin 300072, China

## Abstract

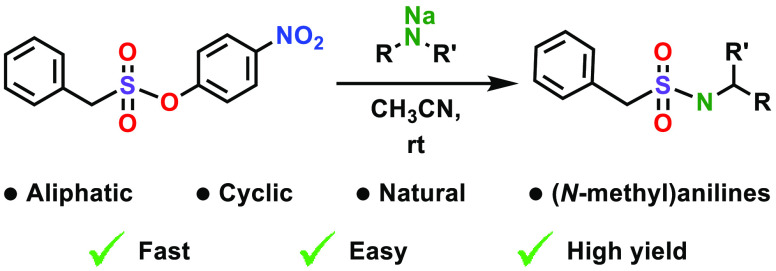

Sulfonamides have many important biological applications, yet their synthesis often involves long reaction times under dry and non-ambient conditions. Here we report the synthesis of a large range of sulfonamides at room temperature using 4-nitrophenyl benzylsulfonate as a starting material. Sulfonamides were prepared from a wide range of aliphatic, linear, and cyclic amines, anilines, and *N*-methylanilines. The yields and reaction times observed here were comparable to or better than those reported previously, establishing sulfur-phenolate exchange as a viable alternative.

The recent (re)discovery and development of the sulfur fluoride exchange (SuFEx) reaction, in which an S–F bond is replaced by an S–O or S–N bond,^1^ has greatly expanded the range of sulfonyl-containing compounds that are available to molecular scientists.^2^ As a result, products of the SuFEx reaction can be found in fields ranging from polymer chemistry via organic synthesis to medicinal chemistry.^3−9^ For this last field, sulfonamides in particular are an important class of compounds, with many applications such as anticancer^10^ or antiviral^11^ drugs, and protein^12−14^ or enzyme^15−19^ inhibitors. It is therefore not surprising that there has been much research into the synthesis of sulfonamides. Currently, the most common synthesis pathway is the reaction of the desired sulfonyl chloride with the desired amine under dry and basic conditions. While the yields obtained using this method are generally good, long reaction times,^20^ (microwave) heating, and/or non-ambient conditions^16,17,21^ are often needed, unless the amine is first activated by the addition of a lithium agent.^22,23^ Apart from this, the sulfonyl chlorides used as starting material generally display poor hydrolytic stability, leading to lower yields when the reaction medium is not thoroughly dried. While there are examples of the use of other leaving groups, such as thiazoles^24^ or phosphates,^25^ these routes suffered from poor yields. Sulfonyl fluorides can also be used as starting materials, and indeed show good yields and higher stability against hydrolysis than the corresponding sulfonyl chlorides.^26^ However, for such use of sulfonyl fluorides, elevated temperatures or catalysts are often needed.^27−30^ Furthermore, there is an environment-driven trend to limit the use of fluorine-containing chemicals in industry. As a result, there is clearly a need for fluorine-free alternative starting materials that are easy to make, more stable than sulfonyl chlorides, and still able to produce sulfonamides in good yields without catalysts in <12 h of reaction time.

Previously, we have shown that the *p*-nitrophenolate moiety can function as an excellent leaving group in S(VI) exchange chemistry, making the corresponding sulfur-phenolate exchange (SuPhenEx) an efficient, fluorine-free alternative for the SuFEx reaction.^31,32^ Specifically, 4-nitrophenyl benzylsulfonate (**1**) was shown to be a good alternative to benzylsulfonyl chloride or fluoride as a starting material, by the creation of a large library of sulfonates in a near-quantitative fashion using a simple and fast SuPhenEx reaction at room temperature.^32^ Now, we demonstrate that the same class of starting materials can also be used to create a large array of sulfonamides in good yields via the reaction with a wide range of alkylamines and *N*-alkylated anilines (Figure 1).

**Figure 1:**
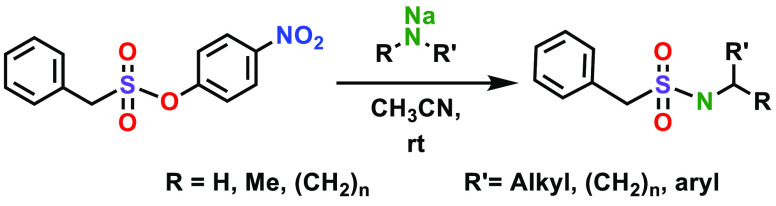
Overview of the SuPhenEx reaction with amines.

To this end, we first allowed **1** to exchange with aliphatic amines, using different primary butylamines as model compounds, and—as in the entire current study—NaH to create the corresponding N-centered anion (Table 1). Briefly, NaH was added to the amine, and the mixture was stirred for 5 min, after which **1** was added (see the Supporting Information for more details). All of the primary alkylamines reacted quantitatively within 5 min to form the corresponding sulfonamides. While even the *tert*-butyl isomer (product **3c**) reacted readily, a significant increase of the reaction time to 2 h for quantitative product formation (all still at rt) was observed upon reaction of **1** with *N*-methylbutylamine (**2d**). For the corresponding *N*-ethyl derivative, yielding product **3e**, the reaction time increased to 6 h, though without compromising the yield. These results are in line with literature, where similarly high yields were obtained for aliphatic amines reacting with benzylsulfonyl chloride, though typically after longer reaction times (2–19 h).^10,18^

**Table 1:**
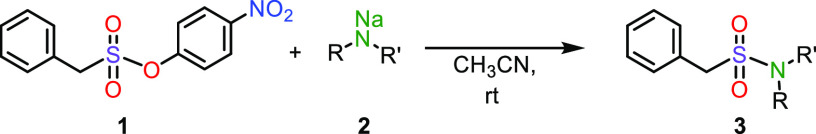

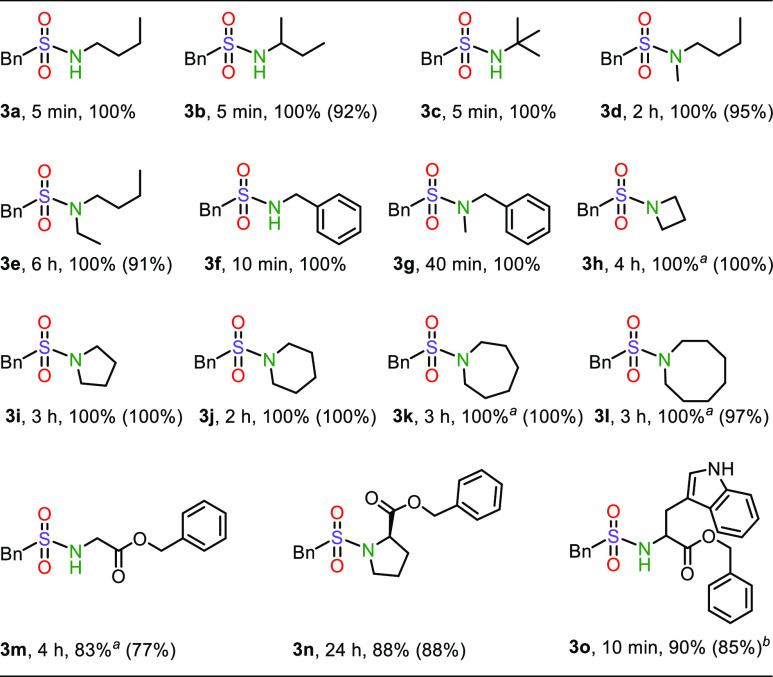
SuPhenEx Reaction of **1** with Linear and Cyclic Aliphatic Amines^c^

a The NMR yield was confirmed using an internal standard.

b 2 equiv of NaH was used.

c Yields were determined by ^1^H NMR measurements. Isolated yields are reported in parentheses. Reaction times refer to the time needed for full conversion of **1**. Reaction conditions: 0.20 mmol of **1** and 1.1 equiv of **2** in 0.6 mL of CH_3_CN.

For benzylic amines, the effect of an extra substituent on the amine was less pronounced, as demonstrated by the reaction times of **3f** and **3g**. In literature, no comparable reactions in solution are known, but 4-iodobenzylamine required 2 h to be coupled to surfaces functionalized with sulfonyl fluoride,^33^ while amines with two benzylic groups were coupled to benzylsulfonyl chloride in <86% yield after an overnight reaction.^34^ The reaction times found here (10–40 min at rt) thus show a considerable improvement on these previous results.

After determining the effect of additional substitutions on the reactivity of amines, we turned our attention to the effects of ring size and ring strain by performing the exchange reaction with simple cyclic amines ranging from four- (azetidine) to eight-membered (azocane) rings as model compounds (yielding products **3h**–**3l**). The highest reactivity was found for piperidine (**2j**), with a reaction time of 2 h. This is in line with literature, where the basicity and charge on the N atom of piperidine are calculated—and the basicity is also experimentally determined—to be the highest among all cyclic amines studied here.^35^ This is a direct effect of the low ring strain in piperidine; as the ring strain increases, the orbitals forming the N–C bonds are forced to increase in p character, which leads the orbitals involved in the N–H bond and nitrogen lone pair to increase in s character. As the s orbital is closer to the nucleus, this stabilizes the N–H bond and nitrogen lone pair, thereby lowering the basicity of the amine. In piperidine, the N–C bonds are closest to the natural angles of the p orbitals, which explains the higher reactivity compared to both smaller and larger rings. In previous studies using six-membered cyclic amines and benzylsulfonyl chloride, yields were either lower (60–90%) when the reaction was performed at 0–25 °C^13,14,36^ or equal to the yield obtained here when the reaction was performed under reflux conditions.^37^ The combination of reduced reaction times, quantitative yields, and removal of the need for ultradry conditions shows the SuPhenEx reaction to be at least a viable alternative to these previous methods.

The previously demonstrated stability of **1** in aqueous solutions^32^ is, of course, especially effective in the SuPhenEx reactivity for use in many biological applications, specifically those involving polar natural amines (Table 1). To demonstrate its scope, we thus used several amino acids to attach to **1**. Since previous studies in our lab showed an incompatibility of carboxylic acid groups with the SuPhenEx reaction,^32^ a benzyl protecting group was used on the C-terminus of the amino acids. With this precaution, it was possible to obtain all three products **3m**–**o** in good yield, *i.e*., comparable to but minimally slightly faster than previously reported coupling reactions of benzylsulfonyl chloride to (benzyl-protected) amino acids.^20,38^ The longer reaction times for glyine (**3m**) and l-proline (**3n**), for which reaction times of minutes (glycine) or a couple of hours (l-proline) were expected, can be explained by the low solubility of these more polar amines in acetonitrile. Indeed, when 1 equiv of 15-crown-5 was added during the reaction, both products **3m** and **3n** were obtained in <30 min.

After these first studies, we tested a series of anilines and *N*-methylanilines (Table 2). Here, as opposed to the trend observed in Table 1, the reactivity was increased for secondary amines with respect to primary amines, and a wide variety of *N*-methylanilines reacted in good yields (83–100%) within 2 h. Only two *N*-methylanilines did not react well: *N*-methyl-4-nitroaniline (**4f**), with the same strongly electron-withdrawing substituent as the phenolate leaving group in **1**, gave no more than trace amounts of product, and the product from *p*-NH_2_-substituted *N*-methylaniline **5g** was obtained in low yield as well due to side reactions with the additional amine moiety on this molecule. Interestingly, the only SuPhenEx product that was observed was the one with attachment via the *N*-methylamine group, demonstrating the difference in reactivity between anilines and *N*-methylanilines.

**Table 2:**
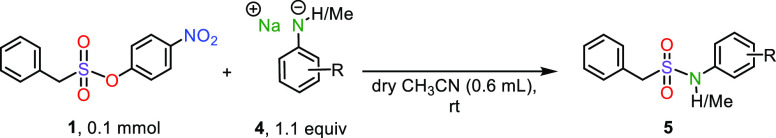

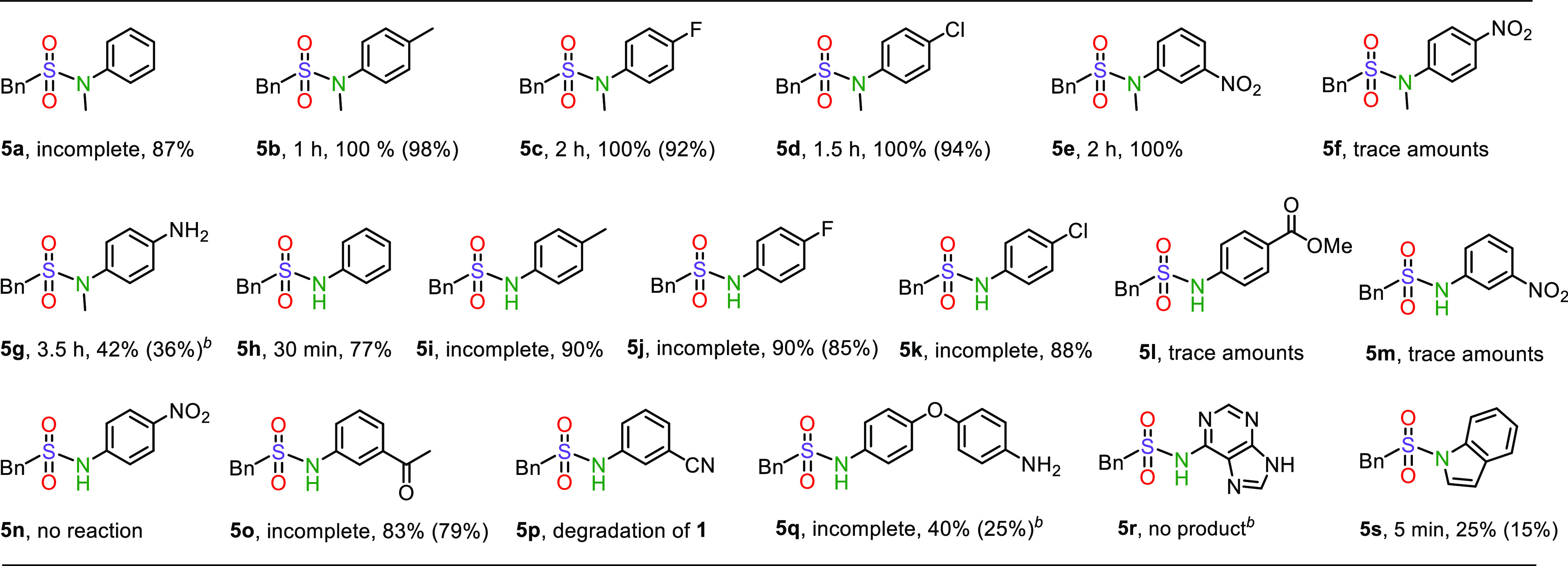
SuPhenEx Reaction of **1** with *N*-Methylanilines and Anilines^a^

a Yields were determined by ^1^H NMR measurements after filtration through a short silica plug. Isolated yields are reported in parentheses. Incomplete reactions were stopped after 5 days.

b 2 equiv of NaH was used.

For the primary anilines, only aniline itself gave full conversion, although good yields were still obtained for anilines with electronically moderate substituents (**5h**–**k**, **5o**) after longer reaction times than observed for *N*-methylanilines. When a stronger electron-withdrawing substituent was attached to the aniline, *e.g.*, a nitro (**5m**, **5n**) or cyano (**5p**) group, only trace amounts of product were found, or the starting material **1** degraded before product was formed. This is in line with previous room-temperature methods using benzylsulfonyl chloride as a starting material,^39−42^ although Cheng et al. reported the formation of a dinitro product in 89% yield using a 50% excess of aniline and base.^41^ Higher yields have also been reported for anilines using a microwave reaction at 130 °C.^16,17,21^ For **5r**, which is stabilized by resonance after deprotonation, no product was found, while the sterically hindered and strained indole **5s** gave a low yield of 25% product yet had a surprisingly short reaction time, comparable to those of aliphatic primary amines. As a result of the reduced reaction rate, the SuPhenEx reactions with anilines were also more susceptible to water. When the reaction vial was opened to air during a test reaction, the observed yield was lower, and more degradation of **1** could be observed. At the same time, when the reaction was performed under completely dry—but not oxygen-free—conditions, product formation took significantly longer, showing that trace amounts of water do actually seem to favor the reaction. Analysis of a crystalline side product found after degradation indicated hydrolysis of **1**. ^1^H NMR analysis of the crude reaction mixture after degradation also indicated side reactions on the amine itself, though the exact products were not analyzed in detail. As the corresponding reaction with phenols showed no such dependence on the presence of water,^32^ we postulate that a reaction occurs between the deprotonated amine and water. As a result of this, the amount of amine available for reaction with **1** is decreased, causing a lower yield and eventual base-catalyzed hydrolysis of **1**.

Next, **1** was reacted with other nitrogen-based nucleophiles, such as hydroxylamines, a hydrazine, a hydrazide, and an amide (Table 3), and some interesting results were found: whereas *O*,*N*-dimethylhydroxylamine (**6a**) gave 100% yield, the NH_2_-bearing hydroxylamine **6b** gave only trace amounts of product. Similarly, substituted hydrazine **6c** gave full conversion to the desired product, while hydrazide **6d** gave only 30% yield after 5 days. For amide **6e**, the negative charge on the nitrogen atom is stabilized by the carbonyl group, preventing product formation and leading to the eventual degradation of **1**.

**Table 3:**
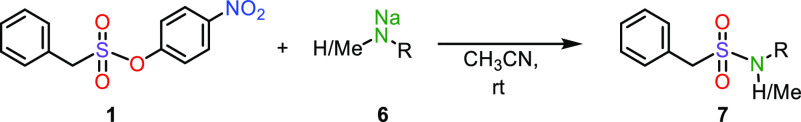

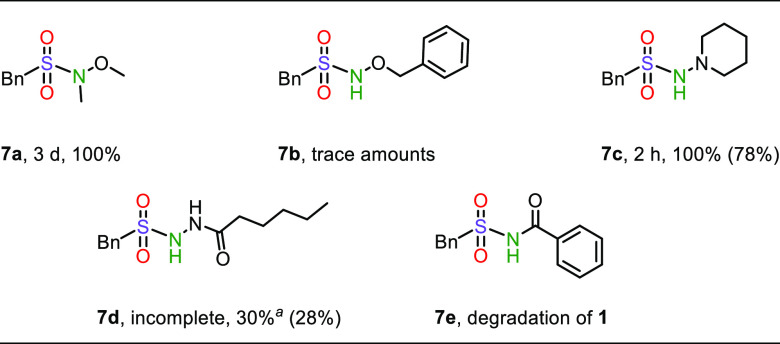
SuPhenEx Reaction of **1** with Other Nitrogen-Containing Nucleophiles^b^

a NMR yield confirmed using an internal standard.

b Yields were determined by ^1^H NMR measurements. Isolated yields are reported in parentheses. Reaction conditions: 0.20 mmol of **1** and 1.1 equiv of **2** in 0.6 mL of CH_3_CN. Incomplete reactions were stopped after 5 days.

Finally, to further investigate the scope of the sulfonamide-forming SuPhenEx reaction, we varied the leaving group to a series of other phenolic moieties with less electron-withdrawing substituents at the *para*-position, and to a non-phenolic leaving group, namely, the 2-butoxy moiety. To this aim, we repeated the SuPhenEx reaction with butylamine (**2a**) with several alternative starting materials (Table 4). Changing the substituent on the phenolate leaving group from 4-nitro to 4-cyano led to a small increase in reaction time, from 5 to 15 min. When a trifluoromethyl group was used instead, the yield of **3a** decreased to 85%, while for the unsubstituted phenolate, the yield decreased even further, to just 40%. Finally, when an aliphatic 2-butoxy leaving group was used, no conversion was observed at all. This demonstrates that the reactivity of the SuPhenEx reaction can gradually be tuned by the substituent on the leaving group.

**Table 4:**
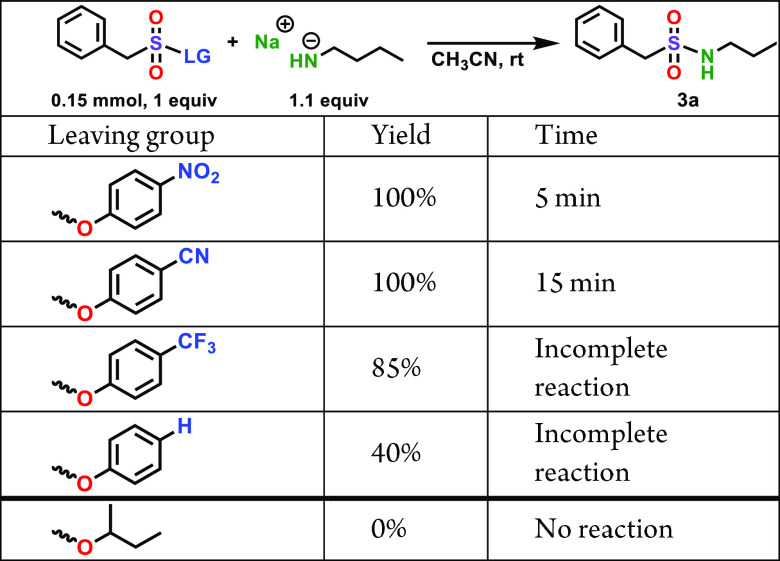
Effect of the Leaving Group on the Reactivity of the SuPhenEx Reaction with Butylamine

After determining the scope of the SuPhenEx reaction for the synthesis of sulfonamides, we investigated the degree of exchange with a good O-centered nucleophile. This is relevant, because for O-centered nucleophiles, we recently discovered the SuPhenEx reaction to be a dynamic covalent reaction with, *e.g.*, potential for controlled polymer degradation.^31^ To this end, we selected three sulfonamide products (**3b**, **3k**, and **5b**) and reacted them with sodium 4-methoxyphenolate, a strong oxygen-based nucleophile (Scheme 1). For all three reactions, no conversion or degradation of the sulfonamides was observed after 6 days of reaction time, and the starting products **3b**, **3k**, and **5b** were recovered quantitatively. This high stability of the sulfonamide products under these reaction conditions thus adds to the features of the SuPhenEx reaction in multistep synthesis.

**Scheme 1:**
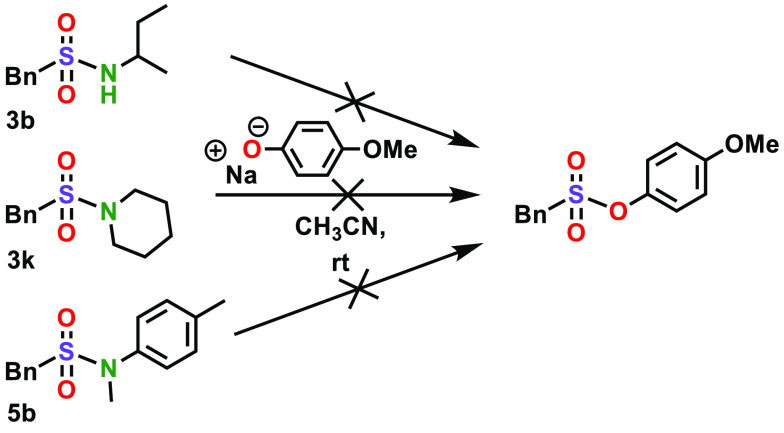
Sulfonamides Remain Stable under SuPhenEx Conditions with a Strong Nucleophile

In conclusion, we have demonstrated that the SuPhenEx reaction is a powerful S(VI) exchange chemistry alternative to conventional synthesis methods for the production of sulfonamides, using a stable and easy-to-use starting material. The reaction works well for a wide range of linear and cyclic aliphatic amines, (C-protected) amino acids, and *N*-alkylanilines. Yields obtained with the SuPhenEx reaction are comparable to or higher than those reported previously, while reaction times are often shorter and no rigorous drying is needed. Finally, we demonstrated the stability of the created sulfonamides toward nucleophilic attack, allowing the SuPhenEx reaction to be used in orthogonal syntheses. With all this, we hope to expand the synthetic toolbox available to (bio)molecular scientists.

## Data Availability

All underlying data are available in the article itself and its Supporting Information.

## Supporting Information Available

Experimental details and characterization of novel compounds (PDF)
