# Correction to “Sulfur-Phenolate Exchange as a Mild, Fast, and High-Yielding Method toward the Synthesis of Sulfonamides”

**DOI:** 10.1021/acs.orglett.3c00361

**Published:** 2023-02-20

**Authors:** Alyssa F. J. van den Boom, Han Zuilhof

In both the TOC and Abstract graphics and in Figure 1 a carbon atom was accidently added in the product, as if an amine anion R-N(-)-R′ had been turned into HN(-)-CHRR′. This error has been corrected in the graphics below.
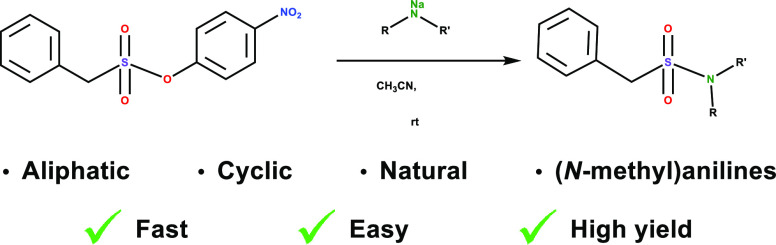


**Figure 1:**
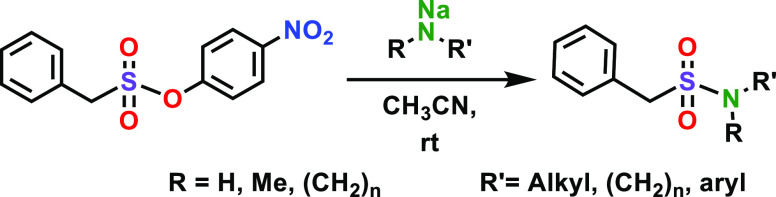
Overview of the SuPhenEx reaction with amines.

