# Supplementary material for: Sulfur-Phenolate Exchange as a Mild, Fast, and High-Yielding Method toward the Synthesis of Sulfonamides
Source: Org Lett. 2023 Jan 31;25(5):788–93. doi: 10.1021/acs.orglett.2c04292 (PMC9926510; doi:10.1021/acs.orglett.2c04292)
Supplement: Supplementary file 1 — ol2c04292_si_001.pdf [file ol2c04292_si_001.pdf]

Supporting information

Sulphur-Phenolate Exchange as a Mild, Fast and High-Yielding Method towards the Synthesis of Sulfonamides

Alyssa F.J. van den Boom[a] and Han Zuilhof*[a,b]

[a] Laboratory of Organic Chemistry, Wageningen University, Stippeneng 4, 6708WE Wageningen, The Netherlands.
[b] School of Pharmaceutical Science and Technology, Tianjin University, 92 Weijin Road, Tianjin 300072, China.

*corresponding author email: han.zuilhof@wur.nl

Contents

General information S2

Chemicals S2

Analysis S2

Synthetic procedures S3

4-Nitrophenyl phenylmethanesulfonate **1** S3

General procedure for the synthesis of products (for NMR screening) S3

Products for isolated yields and full characterization S3

Characterization of compounds S4

NMR spectra of novel compounds S13

References S36

General information

Chemicals

Sodium hydride (60% dispersion in mineral oil), 4-nitrophenol, phenylmethylsulfonyl fluoride, aniline, benzylamine, *n*-butylamine, *sec*-butylamine, *tert*-butylamine, *N*-ethylbutylamine, glycine benzyl ester hydrochloride, *L*-proline benzyl ester, *L*-tryptophan benzyl ester, piperidine, 3-nitro-*N*-methylaniline, *N*-methyl-4-nitroaniline, *p*-toluidine, 4-fluoroaniline, 4-chloroaniline, 3'-aminoacetophenone, 3-nitroaniline, adenine, 3-aminobenzonitrile, methyl 4-aminobenzoate, 4-nitroaniline, phenol, 2-butanol, 4-cyanophenol, 4-(trifluoromethyl)phenol were bought from Merck Life Science N.V.; acetonitrile-d_3 was bought from Fisher Scientific B.V.; 4-(methylamino)benzoate was bought from Alfa Aesar; *N*-1-methylbenzene-1,4-diamine dihydrochloride was bought from Fluorochem Ltd; *N*-methyl-1-butylamine, 1-aminopiperidine, 1-cyclohexylhydrazine hydrochloride, hexanehydrazine, *O*-benzylhydroxylamine, *O,N*-dimethyl-hydroxylamine hydrochloride, benzamide, *N*-methyl-*p*-toluidine, 4-chloro-*N*-methylaniline, 4-fluoro-*N*-methylaniline, heptamethyleneimine were bought from ABRC GmbH; azetidine, pyrrolidine, indole, *N*-methylbenzylamine, *N*-methylaniline, hexamethyleneimine, 4,4'-diaminodiphenyl ether were bought from TCI Europe N.V. All chemicals were used as received. Phenyl benzylsulfonate, 2-butoxy benzylsulfonate, 4-cyanophenyl benzylsulfonate, and 4-(trifluoromethyl)phenyl benzylsulfonate were prepared as described in [1].

Analysis

NMR measurements were conducted on a 400 MHz Bruker Avance III at 298K, and the resulting data were analyzed using MestReNova software, version 14.1.0-24037. Spectra were calibrated relative to signals corresponding to the non-deuterated solvents (CH_3CN solvent peak) – at 1.94 ppm for ^{1}H spectra, and 1.32 ppm for ^{13}C spectra. Spectra recorded in $CDCl_3$ were calibrated on the non-deuterated solvent peak at 7.26 ppm (^{1}H spectra) and 77.16 ppm (^{13}C spectra). ^{19}F spectra were not calibrated. Abbreviations used in the description of NMR data are as follows: chemical shift (δ = ppm), multiplicity (s = singlet, d = doublet, t = triplet, q = quartet, p= pentet, sextet = sext, h = heptet, dt = doublet of triplets, m = multiplet, br = broadened), coupling constant (*J*, Hz).

High-resolution mass spectra (HRMS) were recorded on a Thermo Scientific Exactive 1.1 with an orbitrap mass analyzer, using a DART gun from Ion Sense. The temperature of the DART gun was set to 300-550 °C, with a nitrogen gas flow. Data were analyzed using Thermo Xcalibur software, version 2.2 SP1.48.

IR spectra were recorded on a Bruker Tensor 27 spectrometer equipped with a diamond ATR accessory (64 scans; 4 cm^{-1} resolution; range 4000-350 cm^{-1}). Strong or indicative peaks in the region 4000-1000 cm^{-1} are mentioned for each novel compound.

TLC analysis was performed on pre-coated, alumina-backed silica gel plates. TLC plates were analyzed by UV fluorescence (254 nm) or $KMnO_4$ stain followed by heating.

Column chromatography for isolated yields and full characterization was performed using silica (40-63 µm, 230-400 mesh) and 35-60% ethyl acetate in hexane, depending on the polarity of the product. To perform flash chromatography, a connector connected to a compressed-air flow was installed on the column.

Synthetic procedures

All reactions were performed at room temperature, under ambient atmosphere.

4-Nitrophenyl phenylmethanesulfonate 1

NaH (60% in oil, 293 mg, 7.34 mmol, 1.4 equiv) was added to 4-nitrophenol (948 mg, 6.82 mmol, 1.3 equiv) in 80 ml of dry tetrahydrofuran, and the solution was stirred for 5 min. Next, phenylmethanesulfonyl chloride (1.0 g, 5.25 mmol, 1.0 equiv) was added, and stirring was continued for 24 h. Then, the reaction mixture was diluted with 150 ml of DCM and transferred to a separatory funnel, where it was washed with water (3 × 150 ml). The organic phase was isolated, dried over sodium sulphate, and the solvent was evaporated. Next, the product was further purified by column chromatography (n-hex/EA) (using a Biotage® system and SiliCycle® precast silica columns (200−300 mesh or 300−400 mesh)) to yield compound **1** (1.37 g, 89%) as white crystals.

General procedure for the synthesis of products (for NMR screening)

In a typical exchange reaction, 5 mg of NaH (0.124 mmol, 1.2 equiv) and 0.113 mmol (1.1 equiv) of aniline/amine were dissolved in 0.9 mL of CH_3CN (stored over $NaSO_4$), and the reaction was stirred for ~5 min to generate the deprotonated amine. After this time, 30 mg (0.103 mmol, 1.0 equiv) of **1** was added, and stirring was continued. TLC (35% ethyl acetate in hexane) was used to monitor the progress of the reaction by the disappearance of the spot belonging to 4-nitrophenyl phenylmethanesulfonate. When necessary, staining with a standard permanganate stain was performed to better visualize spots. When the spot for **1** had fully disappeared, the reaction mixture was filtered through a short silica plug to remove the sodium nitrophenolate. The solvents were evaporated, and the mixture was directly analyzed using ^{1}H NMR. No internal standard was needed in this case, as the sodium nitrophenolate was filtered off, and no other side products – except in the case of degradation – are formed during the reaction. As a result, the reaction mixture only contains the product and any remaining starting material, both of which provide well-separated signals in ^{1}H NMR. The yield could therefore be determined by the disappearance of the signals belonging to 4-nitrophenol, with a simultaneous appearance of product signals. A reference spectrum of the reactant amine was recorded in all cases, and successful binding was further confirmed by a change in chemical shift for the signals belonging to the added amine upon attachment to the S(VI) hub. When the reaction was filtered prematurely – or when conversion was <100% – signals from an attached nitrophenol group were still visible (though with a lower integral value), and the product signals of the partially attached amine had lower integrals than expected based on full conversion. When degradation took place, no product signals were observed, and signals from the starting material had shifted or disappeared. To confirm the accuracy of this method, an internal standard (vinyltrimethylsilane) was added to several reaction mixtures after evaporation of the solvent, and the yield was determined both with and without internal standard. The difference in yield determined in this way was <1%, confirming the accuracy of the NMR yields determined without internal standard.

Products for isolated yields and full characterization

Compounds for which the isolated yield was determined, and novel compounds that had to be characterized more extensively, were synthesized in the same way as described above for the NMR products, on a scale of 60 mg (0.21 mmol) of **1**. After the reaction was completed, the reaction mixture was transferred to an extraction funnel, diluted with ~15 ml of ethyl acetate, and washed with ~4 ml fractions of water, until the water layer was colorless (typically 5 fractions were needed). Then, the organic layer was washed 3x with ~4 ml of an 1M aqueous solution of $KHSO_4$. The organic layer was isolated, dried with Na_2SO_4, filtered, and concentrated on a rotavapor. The crude product was further purified using flash column chromatography (silica gel) with 35%-60% ethyl acetate in hexane, depending on the polarity of the product. A representative example of the synthesis of 1-(benzylsulfonyl)azepane (**3k**) on a scale of 0.31 mmol **1** is provided below.

3k: 1-(benzylsulfonyl)azepane

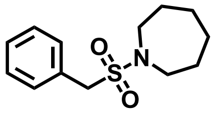

49 mg (60% dispersion, 1.2 mmol, 1.2 equiv) NaH was weighed into a 10 mL roundbottom flask equipped with a stir bar, and 109 mg (1.1 mmol, 1.1 equiv) hexamethyleneimine was added. Next, 6 ml of acetonitrile – stored over sodium sulfate – was added, and the resulting mixture was stirred for 5 min. 293 mg (1.0 mmol, 1 equiv) of 4-nitrophenyl phenylmethanesulfonate **1** was added, and the reaction was allowed to stir at room temperature. Reaction progress was monitored by TLC (35% ethyl acetate in hexane), and full conversion was confirmed by the disappearance of the spot belonging to **1**. After 3 h, this spot had fully disappeared, and the reaction mixture was transferred to an extraction funnel along with 60 ml of ethyl acetate. The organic layer was washed 5× with 15 ml of water, until the water layer was completely colorless. The organic layer was collected, dried over sodium sulfate, and concentrated on a rotavapor. The product was further purified by flash column chromatography (silica gel, 35% ethyl acetate in hexane) to yield 253 mg (1.0 mmol, 100%) of **3k** as an off-white solid.

Characterization of compounds

3a: *N*-butyl-1-phenylmethanesulfonamide

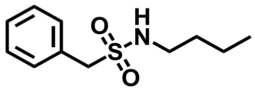

Prepared according to the general procedure described above using butylamine as the amine. NMR yield: 100%

HRMS (DART): *m/z*: $[M+H]^+$, calculated for $C_{11}H_{18}NO_2S^+$: 228.1053; found: 228.1053.

^{1}H NMR (CD_3CN, 400 MHz): δ 7.44 – 7.33 (m, 5H), 5.10 (s, 1H), 4.25 (s, 2H), 2.97 (q, *J* = 7.0 Hz, 2H), 1.45 (p, *J* = 7.0 Hz, 2H), 1.32 (h, *J* = 7.0 Hz, 2H), 0.89 (t, *J* = 7.3 Hz, 3H).

These values match previously reported data.[2]

3b: *N*-(*sec*-butyl)-1-phenylmethanesulfonamide

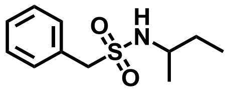

Prepared according to the general procedure described above using *sec*-butylamine as the amine. Purified by column: 35% ethyl acetate in hexane. White crystals. NMR yield: 100%; isolated yield: 42.6 mg, 92%.

HRMS (DART): *m/z*: $[M+H]^+$, calculated for $C_{11}H_{18}NO_2S^+$: 228.1053; found: 228.1052.

^{1}H NMR (CD_3CN, 400 MHz): δ 7.49 – 7.33 (m, 5H), 5.01 (d, *J* = 7.8 Hz, 1H), 4.26 (s, 2H), 3.28 (dh, *J* = 8.1, 6.6 Hz, 1H), 1.61 – 1.36 (m, 2H), 1.14 (d, *J* = 6.6 Hz, 3H), 0.89 (t, *J* = 7.4 Hz, 3H). **^{13}C NMR (CD_3CN, 101 MHz):** δ 131.4, 131.0, 129.0, 128.8, 117.9, 59.5, 52.1, 30.8, 21.3, 10.1.

$\tilde{\nu}_{max}$: 3284, 3066, 2995, 2966, 2924, 2875, 1492, 1456, 1429, 1298, 1265, 1157, 1109 cm^{-1}

3c: *N*-(*tert*-butyl)-1-phenylmethanesulfonamide

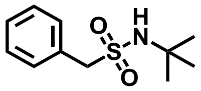

Prepared according to the general procedure described above using *tert*-butylamine as the amine. NMR yield: 100%

HRMS (DART): *m/z*: $[M+H]^+$, calculated for $C_{11}H_{18}NO_2S^+$: 228.1053; found: 228.1053.

^{1}H NMR (CD_3CN, 400 MHz): δ 7.47 – 7.32 (m, 5H), 4.93 (s, 1H), 4.26 (s, 2H), 1.33 (s, 9H).

These values match previously reported data.[3]

3d: *N*-butyl-*N*-methyl-1-phenylmethanesulfonamide

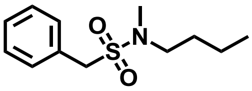

Prepared according to the general procedure described above using *N*-methylbutylamine as the amine. Purified by column: 35% ethyl acetate in hexane. Light yellow crystals. NMR yield: 100%; isolated yield: 46.9 mg, 95%.

HRMS (DART): *m/z*: $[M+H]^+$, calculated for $C_{12}H_{20}NO_2S^+$: 242.1209; found: 242.1210.

^{1}H NMR (CD_3CN, 400 MHz): δ 7.48 – 7.30 (m, 5H), 4.24 (s, 2H), 3.01 (t, *J* = 7.2 Hz, 2H), 2.73 (s, 3H), 1.47 (p, *J* = 7.3 Hz, 2H), 1.27 (h, *J* = 7.3 Hz, 2H), 0.89 (t, *J* = 7.4 Hz, 3H). **^{13}C NMR (CD_3CN, 101 MHz):** δ 131.9, 130.9, 129.5, 129.3, 118.3, 55.9, 50.7, 35.2, 30.8, 20.3, 13.9.

$\tilde{\nu}_{max}$: 3063, 3034, 2956, 2929, 2870, 1603, 1495, 1456, 1317, 1205, 1144, 1086, 1018 cm^{-1}

3e: *N*-butyl-*N*-ethyl-1-phenylmethanesulfonamide

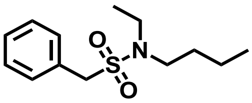

Prepared according to the general procedure described above using *N*-ethylbutylamine as the amine. Purified by column: 35% ethyl acetate in hexane. Thick yellowish oil. NMR yield: 100%; isolated yield: 47.5 mg, 91%.

HRMS (DART): *m/z*: $[M+H]^+$, calculated for $C_{13}H_{22}NO_2S^+$: 256.1366; found: 256.1376.

^{1}H NMR (CD_3CN, 400 MHz): δ 7.44 – 7.33 (m, 5H), 4.22 (s, 2H), 3.14 (q, J = 7.1 Hz, 2H), 3.05 (t, J = 7.6 Hz, 2H), 1.48 (p, J = 7.8 Hz, 2H), 1.27 (h, J = 7.4 Hz, 2H), 1.10 (t, J = 7.1 Hz, 3H), 0.90 (t, J = 7.4 Hz, 3H). **^{13}C NMR (CD_3CN, 101 MHz):** δ 131.9, 131.2, 129.4, 129.2, 57.7, 48.5, 43.9, 32.1, 20.5, 15.3, 14.0.

$\tilde{\nu}_{max}$: 3064, 3034, 2958, 2931, 2874, 1595, 1497, 1456, 1331, 1192, 1146, 1122, 1024 cm^{-1}

3f: *N*-benzyl-1-phenylmethanesulfonamide

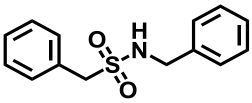

Prepared according to the general procedure described above using benzylamine as the amine. NMR yield: 100%.

HRMS (DART): *m/z*: $[M+H]^+$, calculated for $C_{14}H_{16}NO_2S^+$: 262.0896; found: 262.0888.

^{1}H NMR (CD_3CN, 400 MHz): δ7.43 – 7.26 (m, 10H), 5.62 (s, 1H), 4.26 (s, 2H), 4.17 (d, *J* = 6.4 Hz, 2H).

This compound was first reported by Johnson *et al*. (1914).[4]

3g: *N*-benzyl-*N*-methyl-1-phenylmethanesulfonamide

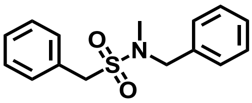

Prepared according to the general procedure described above using *N*-methylbenzylamine as the amine. NMR yield: 100%.

HRMS (DART): *m/z*: $[M+H]^+$, calculated for $C_{15}H_{18}NO_2S^+$: 276.1053; found: 276.1052.

^{1}H NMR (CD_3CN, 400 MHz): δ 7.46 – 7.39 (m, 5H), 7.39 – 7.33 (m, 2H), 7.33 – 7.27 (m, 3H), 4.34 (s, 2H), 4.17 (s, 2H), 2.64 (s, 3H).

This product was previously reported in Nikam *et al*. (1998).[5]

3h: 1-(benzylsulfonyl)azetidine

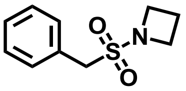

Prepared according to the general procedure described above using azetidine as the amine. Purified by column: 35% ethyl acetate in hexane. White solid. NMR yield: 100%; NMR yield (IS): 100%; isolated yield: 43.2 mg, 100%.

HRMS (DART): *m/z*: $[M+H]^+$, calculated for $C_{10}H_{14}NO_2S^+$: 212.0740; found: 212.0738.

^{1}H NMR ($CDCl_3$, 400 MHz): δ 7.45 – 7.40 (m, 2H), 7.41 – 7.33 (m, 3H), 4.19 (s, 2H), 3.78 (t, *J* = 7.7 Hz, 4H), 2.15 (p, *J* = 7.8 Hz, 2H). **^{13}C NMR ($CDCl_3$, 101 MHz):** δ 130.9, 128.9, 128.82, 128.77, 57.7, 51.0, 15.3.

$\bar{\upsilon}_{max}$: 3059, 3028, 2978, 2906, 1593, 1493, 1454, 1335, 1309, 1288, 1234, 1207, 1149, 1126, 1065, 1030 cm^{-1}

3i: 1-(benzylsulfonyl)pyrrolidine

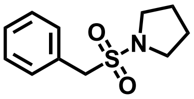

Prepared according to the general procedure described above using pyrrolidine as the amine. Purified by column: 35% ethyl acetate in hexane. Light yellow solid. NMR yield: 100%; isolated yield: 46.1 mg, 100%.

HRMS (DART): *m/z*: $[M+H]^+$, calculated for $C_{11}H_{16}NO_2S^+$: 226.0896; found: 226.0893.

^{1}H NMR (CD_3CN, 400 MHz): δ 7.45 – 7.36 (m, 5H), 4.27 (s, 2H), 3.22 – 3.14 (m, 4H), 1.84 (t, *J* = 3.5 Hz, 2H), 1.83 (t, *J* = 3.6 Hz, 2H).

These values match previously reported values for this compound.[6]

3j: 1-(benzylsulfonyl)piperidine

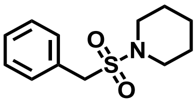

Prepared according to the general procedure described above using piperidine as the amine. Purified by column: 35% ethyl acetate in hexane. Light yellow solid. NMR yield: 100%; isolated yield: 49 mg, 100%.

HRMS (DART): *m/z*: $[M+H]^+$, calculated for $C_{12}H_{18}NO_2S^+$: 240.1053; found: 240.1049.

^{1}H NMR (CD_3CN, 400 MHz): δ 7.45 – 7.34 (m, 5H), 4.20 (s, 2H), 3.18 – 3.07 (m, 4H), 1.58 – 1.47 (m, 6H).

These values match previously reported values for this compound.[6]

3k: 1-(benzylsulfonyl)azepane

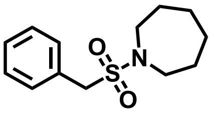

Prepared according to the general procedure described above using hexamethyleneimine as the amine. Purified by column: 35% ethyl acetate in hexane. Off-white solid. NMR yield: 100%; NMR yield (IS): 100%; isolated yield: 51.8 mg, 100%.

HRMS (DART): *m/z*: $[M+H]^+$, calculated for $C_{13}H_{20}NO_2S^+$: 254.1209; found: 254.1207.

^{1}H NMR ($CDCl_3$, 400 MHz): δ 7.36 (s, 5H), 4.22 (s, 2H), 3.08 (t, *J* = 5.5 Hz, 4H), 1.66 – 1.53 (m, 8H). **^{13}C NMR ($CDCl_3$, 101 MHz):** δ 130.7, 129.7, 128.8, 128.6, 57.1, 49.1, 29.9, 26.8.

$\bar{\upsilon}_{max}$: 3063, 3030, 2920, 2850, 1603, 1493, 1446, 1323, 1286, 1225, 1144, 1043 cm^{-1}

3l: 1-(benzylsulfonyl)azocane

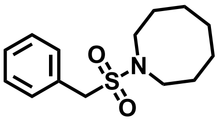

Prepared according to the general procedure described above using heptamethyleneimine as the amine. Purified by column: 35% ethyl acetate in hexane. Light yellow solid. NMR yield: 100%; NMR yield (IS): 100%; isolated yield: 53.1 mg, 97%.

HRMS (DART): *m/z*: $[M+H]^+$, calculated for $C_{14}H_{22}NO_2S^+$: 268.1366; found: 268.1364.

^{1}H NMR (CD_3CN, 400 MHz): δ 7.72 – 7.09 (m, 5H), 4.23 (s, 2H), 3.11 (t, *J* = 5.5 Hz, 4H), 1.69 – 1.50 (m, 10H). **^{13}C NMR (CD_3CN, 101 MHz):** δ 131.8, 131.0, 129.5, 129.2, 55.6, 50.2, 28.8, 27.6, 25.5.

$\bar{\upsilon}_{max}$: 3063, 3034, 2926, 2854, 1495, 1454, 1327, 1149, 1122, 1082, 1028 cm^{-1}

3m: benzyl (benzylsulfonyl)glycinate

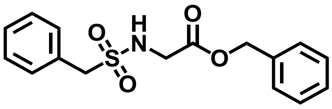

Prepared according to the general procedure described above using glycine benzyl ester as the amine. Purified by extraction with ethyl acetate and water, as described in the general procedure for isolated compounds. White needles. NMR yield: 84%; NMR yield (IS): 83%; isolated yield: 50.3 mg, 77%.

HRMS (DART): *m/z*: $[M+NH_4]^+$, calculated for $C_{16}H_{21}N_2O_4S^+$: 337.1217; found: 337.1218.

^{1}H NMR (CD_3CN, 400 MHz): δ 7.46 – 7.20 (m, 10H), 5.61 (s, 1H), 5.17 (s, 2H), 4.33 (s, 2H), 3.79 (d, *J* = 6.0 Hz, 2H). **^{13}C NMR (CD_3CN, 101 MHz):** δ 170.7, 136.9, 131.9, 130.9, 129.6, 129.5, 129.4, 129.33, 129.25, 67.8, 59.8, 45.3.

$\bar{\upsilon}_{max}$: 3263, 3063, 3036, 2937, 1743, 1593, 1497, 1454, 1419, 1379, 1329, 1309, 1238, 1213, 1198, 1147, 1111, 1026 cm^{-1}

3n: benzyl (benzylsulfonyl)-*L*-prolinate

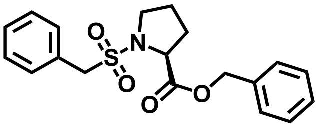

Prepared according to the general procedure described above using *L*-proline benzyl ester as the amine. Purified by recrystallization from 25% ethyl acetate in hexane. Light yellow solid. NMR yield: 88%; isolated yield: 64.7 mg, 88%.

HRMS (DART): *m/z*: $[M+H]^+$, calculated for $C_{19}H_{22}NO_4S^+$: 360.1264; found: 360.1259.

^{1}H NMR (CD_3CN, 400 MHz): δ 7.47 – 7.30 (m, 10H), 5.15 (s, 2H), 4.32 (q, *J* = 13.7 Hz, 2H), 4.17 (dd, *J* = 8.7, 3.6 Hz, 1H), 3.36 – 3.21 (m, 2H), 2.23 – 2.12 (m, 1H), 1.98 – 1.92 (m, 1H), 1.92 – 1.81 (m, 2H). **^{13}C NMR (CD_3CN, 101 MHz):** δ 173.4, 137.1, 131.9, 130.7, 129.6, 129.5, 129.4, 129.3, 129.1, 67.6, 61.8, 57.4, 49.6, 31.7, 25.6.

3o: benzyl (benzylsulfonyl)-*L*-tryptophanate

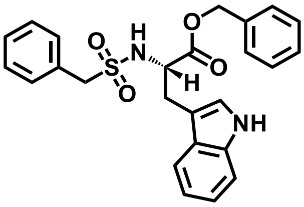

Prepared according to the general procedure described above using 2 equiv of NaH, and *L*-tryptophan benzyl ester as the amine. Purified by recrystallization from 25% ethyl acetate in hexane. Yellow-brown solid. NMR yield: 90%; isolated yield: 78.0 mg, 85%.

HRMS (DART): *m/z*: $[M+NH_4]^+$, calculated for $C_{25}H_{28}N_3O_4S^+$: 466.1795; found: 466.1801.

^{1}H NMR (CD_3CN, 400 MHz): δ 9.17 (s, 1H), 7.49 (d, *J* = 8.1 Hz, 1H), 7.41 (d, *J* = 8.2 Hz, 1H), 7.35 – 7.27 (m, 6H), 7.26 – 7.18 (m, 4H), 7.15 (t, *J* = 7.0 Hz, 1H), 7.10 – 7.01 (m, 2H), 5.62 (d, *J* = 8.7 Hz, 1H), 5.04 (q, *J* = 12.3 Hz, 2H), 4.19 (dt, *J* = 8.7, 6.7 Hz, 1H), 4.14 – 4.05 (m, 2H), 3.23 – 3.10 (m, 2H). **^{13}C NMR (CD_3CN, 101 MHz):** δ 172.8, 137.4, 136.7, 131.8, 130.8, 129.5, 129.4, 129.33, 129.26, 129.2, 125.2, 122.7, 120.1, 119.4, 112.5, 110.0, 67.9, 60.0, 58.2, 29.9.

$\tilde{\nu}_{max}$: 3379, 3284, 3060, 3036, 2935, 2253, 1738, 1497, 1456, 1425, 1333, 1201, 1151, 1126, 1101 cm^{-1}

<u>5a: *N*-methyl-*N*,1-diphenylmethanesulfonamide</u>

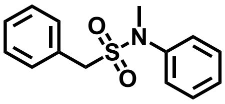

Prepared according to the general procedure described above using *N*-methylaniline as the amine. NMR yield: 87%.

HRMS (DART): *m/z*: $[M+NH_4]^+$, calculated for $C_{14}H_{19}N_2O_2S^+$: 279.1162; found: 279.1161.

^{1}H NMR (CD_3CN, 400 MHz): δ 7.40 – 7.35 (m, 7H), 7.31 – 7.26 (m, 3H), 4.35 (s, 2H), 3.23 (s, 3H).

These values match previously reported data.[6]

<u>5b: *N*-methyl-1-phenyl-*N*-(*p*-tolyl)methanesulfonamide</u>

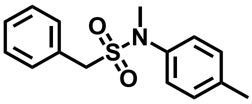

Prepared according to the general procedure described above using *N*-methyl-*p*-toluidine as the amine. Purified by column: 35% ethyl acetate in hexane. White crystals. NMR yield: 100%; isolated yield: 55.2 mg, 98%.

HRMS (DART): *m/z*: $[M+H]^+$, calculated for $C_{15}H_{18}NO_2S^+$: 276.1053; found: 276.1056.

^{1}H NMR (CD_3CN, 400 MHz): δ 7.39 (s, 5H), 7.28 – 7.06 (m, 4H), 4.33 (s, 2H), 3.20 (s, 3H), 2.34 (s, 3H). **^{13}C NMR (CD_3CN, 101 MHz):** δ 140.2, 138.1, 132.0, 130.6, 130.4, 129.6, 129.4, 127.6, 56.0, 39.3, 21.0.

$\tilde{\nu}_{max}$: 3064, 3034, 2926, 1510, 1456, 1342, 1265, 1171, 1144, 1065, 1020 cm^{-1}

<u>5c: *N*-(4-fluorophenyl)-*N*-methyl-1-phenylmethanesulfonamide</u>

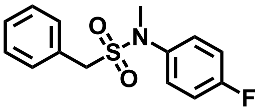

Prepared according to the general procedure described above using 4-fluoro-*N*-methylaniline as the amine. Purified by column: 35% ethyl acetate in hexane. Off-white solid. NMR yield: 100%; isolated yield: 52.6 mg, 92%.

HRMS (DART): *m/z*: $[M+H]^+$, calculated for $C_{14}H_{15}FNO_2S^+$: 280.0802; found: 280.0798.

^{1}H NMR (CD_3CN, 400 MHz): δ 7.39 (s, 5H), 7.31 – 7.25 (m, 2H), 7.15 – 7.06 (m, 2H), 4.35 (s, 2H), 3.21 (s, 3H). **^{13}C NMR (CD_3CN, 101 MHz):** δ 162.2 (d, *J* = 244.6 Hz), 132.0, 130.2, 129.8, 129.7, 129.6, 129.5, 116.6 (d, *J* = 22.9 Hz). **^{19}F NMR (CD_3CN, 376 MHz):** δ 116.62.

$\tilde{\nu}_{max}$: 3074, 2989, 2928, 1504, 1456, 1344, 1217, 1171, 1144, 1057 cm^{-1}

<u>5d: *N*-(4-chlorophenyl)-*N*-methyl-1-phenylmethanesulfonamide</u>

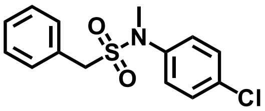

Prepared according to the general procedure described above using 4-chloro-*N*-methylaniline as the amine. Purified by column: 35% ethyl acetate in hexane. White crystals. NMR yield: 100%; isolated yield: 56.9 mg, 94%

HRMS (DART): *m/z*: $[M+H]^+$, calculated for $C_{14}H_{15}ClNO_2S^+$: 296.0507; found: 296.0508.

^{1}H NMR (CD_3CN, 400 MHz): δ 7.66 – 7.30 (m, 7H), 7.24 (dt, *J* = 9.0, 3.0 Hz, 2H), 4.36 (s, 2H), 3.21 (s, 3H). **^{13}C NMR (CD_3CN, 101 MHz):** δ 141.60, 132.75, 131.99, 130.08, 129.93, 129.61, 129.57, 128.72, 56.20, 38.95.

$\tilde{\nu}_{max}$: 3091, 3059, 3055, 2941, 1491, 1456, 1406, 1331, 1267, 1163, 1136, 1090, 1065, 1014 cm^{-1}

5e: *N*-methyl-*N*-(3-nitrophenyl)-1-phenylmethanesulfonamide

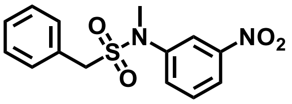

Prepared according to the general procedure described above using 3-nitro-*N*-methylaniline as the amine. NMR yield: 100%.

HRMS (DART): *m/z*: $[M+NH_4]^+$, calculated for $C_{14}H_{18}N_3O_4S^+$: 324.1013; found: 324.1014.

^{1}H NMR (CD_3CN, 400 MHz): δ 8.04 (ddd, *J* = 8.1, 2.2, 1.1 Hz, 1H), 7.98 (t, *J* = 2.2 Hz, 1H), 7.61 (ddd, *J* = 8.2, 2.3, 1.1 Hz, 1H), 7.54 (t, *J* = 8.0 Hz, 1H), 7.38 – 7.35 (m, 5H), 4.44 (s, 2H), 3.30 (s, 3H).

This product was previously reported in Wojciechowski, K. (1997).[7]

5f: *N*-methyl-*N*-(4-nitrophenyl)-1-phenylmethanesulfonamide

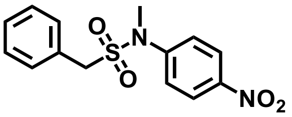

Prepared according to the general procedure described above using *N*-methyl-4-nitroaniline as the amine. Trace amounts.

HRMS (DART): *m/z*: $[M+NH_4]^+$, calculated for $C_{14}H_{18}N_3O_4S^+$: 324.1013; found: 324.1011.

5g: *N*-(4-aminophenyl)-*N*-methyl-1-phenylmethanesulfonamide

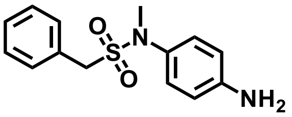

Prepared according to the general procedure described above using 2 equiv of NaH, and *N*1-methylbenzene-1,4-diamine as the amine. Purified by gradient column: 35-60% ethyl acetate in hexane. Green-brownish crystals. NMR yield: 42%; isolated yield: 20.4 mg, 36%.

HRMS (DART): *m/z*: $[M+H]^+$, calculated for $C_{14}H_{17}N_2O_2S^+$: 277.1005; found: 277.1008.

^{1}H NMR (CD_3CN, 400 MHz): δ 7.43 – 7.35 (m, 5H), 7.02 (dt, *J* = 8.8, 3.2 Hz, 2H), 6.61 (dt, *J* = 8.9, 3.1 Hz, 2H), 4.29 (s, 4H), 3.15 (s, 3H). **^{13}C NMR (CD_3CN, 101 MHz):** δ 148.6, 132.00, 131.98, 130.7, 129.5, 129.4, 129.3, 115.4, 55.5, 39.7.

$\tilde{\nu}_{max}$: 3435, 3344, 3051, 2924, 2852, 1622, 1512, 1456, 1336, 1257, 1142, 1061 cm^{-1}

5h: *N*,1-diphenylmethanesulfonamide

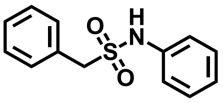

Prepared according to the general procedure described above using aniline as the amine. NMR yield: 77%.

HRMS (DART): *m/z*: $[M+NH_4]^+$, calculated for $C_{13}H_{17}N_2O_2S^+$: 265.1005; found: 265.0996.

^{1}H NMR (CD_3CN, 400 MHz): δ 7.39 – 7.32 (m, 5H), 7.32 – 7.27 (m, 2H), 7.25 – 7.19 (m, 2H), 7.20 – 7.14 (m, 1H), 4.36 (s, 2H).

These values match previously reported data.[8]

5i: 1-phenyl-*N*-(*p*-tolyl)methanesulfonamide

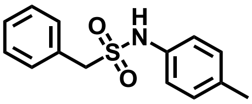

Prepared according to the general procedure described above using *p*-toluidine as the amine. NMR yield: 90%.

HRMS (DART): *m/z*: $[M+NH_4]^+$, calculated for $C_{14}H_{19}N_2O_2S^+$: 279.1162; found: 279.1164.

^{1}H NMR (CD_3CN, 400 MHz): δ 7.40 – 7.34 (m, 3H), 7.33 – 7.26 (m, 2H), 7.17 (d, *J* = 8.0 Hz, 2H), 7.11 (dt, *J* = 8.5, 2.2 Hz, 2H), 4.32 (s, 2H), 2.31 (s, 3H).

This product was previously reported in King *et al.* (1975).[9]

5j: *N*-(4-fluorophenyl)-1-phenylmethanesulfonamide

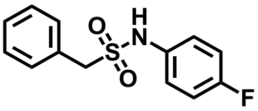

Prepared according to the general procedure described above using 4-fluoroaniline as the amine. Purified by column: 35% ethyl acetate in hexane. White crystals. NMR yield: 90%; isolated yield: 46.1 mg, 85%.

HRMS (DART): *m/z*: $[M+NH_4]^+$, calculated for $C_{13}H_{16}FN_2O_2S^+$: 283.0911; found: 283.0914.

^{1}H NMR (CD_3CN, 400 MHz): δ 7.53 (s, 1H), 7.40 – 7.35 (m, 3H), 7.33 – 7.27 (m, 2H), 7.25 – 7.16 (m, 2H), 7.12 – 7.05 (m, 2H), 4.34 (s, 2H). **^{13}C NMR (CD_3CN, 101 MHz):** δ 162.1, 147.3 (d, *J* = 2485.1 Hz), 132.0, 130.2, 129.6, 129.5, 123.8 (d, *J* = 8.3 Hz), 116.9 (d, *J* = 22.9 Hz), 58.1. **^{19}F NMR (CD_3CN, 376 MHz):** 119.99

$\tilde{\nu}_{max}$: 3261, 3068, 3037, 2929, 1506, 1396, 1327, 1292, 1211, 1147 cm^{-1}

5k: *N*-(4-chlorophenyl)-1-phenylmethanesulfonamide

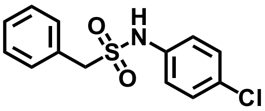

Prepared according to the general procedure described above using 4-chloroaniline as the amine. NMR yield: 88%.

HRMS (DART): *m/z*: $[M+NH_4]^+$, calculated for $C_{13}H_{16}ClN_2O_2S^+$: 299.0616; found: 299.0617.

^{1}H NMR (CD_3CN, 400 MHz): δ 7.38 – 7.35 (m, 3H), 7.33 (dt, *J* = 9.0, 3.1 Hz, 2H), 7.30 – 7.26 (m, 2H), 7.17 (dt, *J* = 9.0, 3.1 Hz, 2H), 4.37 (s, 2H).

This product was previously reported in Marvel *et al*. (1926).[10]

5l: methyl 4-((phenylmethyl)sulfonamido)benzoate

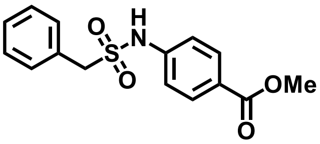

Prepared according to the general procedure described above using methyl 4-aminobenzoate as the amine. Trace amounts.

HRMS (DART): *m/z*: $[M+H]^+$, calculated for $C_{15}H_{16}NO_4S^+$: 306.0795; found: 306.0793.

These values match previously reported data.[11]

5m: *N*-(3-nitrophenyl)-1-phenylmethanesulfonamide

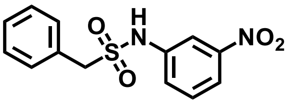

Prepared according to the general procedure described above using 3-nitroaniline as the amine. Trace amounts.

HRMS (DART): *m/z*: $[M+NH_4]^+$, calculated for $C_{13}H_{16}N_3O_4S^+$: 310.0856; found: 310.0858.

5o: *N*-(3-acetylphenyl)-1-phenylmethanesulfonamide

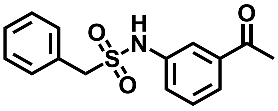

Prepared according to the general procedure described above using 3'-aminoacetophenone as the amine. Purified by column: 40% ethyl acetate in hexane. Light yellow crystals. NMR yield: 83%; isolated yield: 46.8 mg, 79%.

HRMS (DART): *m/z*: $[M+H]^+$, calculated for $C_{15}H_{16}NO_3S^+$: 290.0845; found: 290.0845.

^{1}H NMR (CD_3CN, 400 MHz): δ 7.74 – 7.71 (m, 1H), 7.71 (d, *J* = 1.7 Hz, 1H), 7.46 (t, *J* = 8.2 Hz, 1H), 7.43 – 7.30 (m, 4H), 7.33 – 7.24 (m, 2H), 4.40 (s, 2H), 2.55 (s, 3H). **^{13}C NMR (CD_3CN, 101 MHz):** δ 198.4, 139.5, 139.3, 132.0, 130.7, 130.1, 129.60, 129.58, 125.1, 125.0, 119.9, 58.5, 27.1.

$\tilde{\nu}_{max}$: 3286, 3063, 3036, 2924, 1676, 1589, 1497, 1456, 1406, 1333, 1284, 1257, 1246, 1151, 1130 cm^{-1}

5q: *N*-(4-(4-aminophenoxy)phenyl)-1-phenylmethanesulfonamide

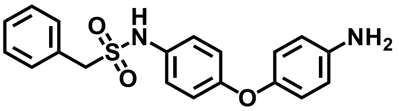

Prepared according to the general procedure described above using 2 equiv of NaH, and 4,4'-diaminodiphenyl ether as the amine. Purified by gradient column: 35-75% ethyl acetate in hexane. White solid. NMR yield: 40%; isolated yield: 18.1 mg, 25%.

HRMS (DART): *m/z*: $[M+2H]^{2+}$, calculated for $C_{19}H_{20}N_2O_3S^{2+}$: 178.0592; found: 178.0589.

^{1}H NMR (CD_3CN, 400 MHz): δ 7.43 (s, 1H), 7.39 – 7.34 (m, 3H), 7.34 – 7.28 (m, 2H), 7.14 (dt, *J* = 8.9, 3.6 Hz, 2H), 6.87 (dt, *J* = 9.1, 3.4 Hz, 2H), 6.81 (dt, *J* = 8.9, 3.4 Hz, 2H), 6.66 (dt, *J* = 8.8, 3.4 Hz, 2H), 4.31 (s, 2H), 4.07 (s, 2H). **^{13}C NMR (CD_3CN, 101 MHz):** δ 157.5, 148.5, 145.7, 132.7, 132.0, 130.4, 129.54, 129.45, 124.1, 121.8, 118.7, 116.5, 57.9.

$\tilde{\nu}_{max}$: 3622, 3394, 3329, 3107, 3047, 2931, 2850, 2679, 1606, 1495, 1317, 1213, 1136, 1011 cm^{-1}

5s: 1-(benzylsulfonyl)-1*H*-indole

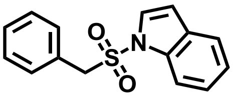

Prepared according to the general procedure described above using indole as the amine. Very light orange/brown solid. This product is difficult to separate from the left-over indole after the reaction. Purified by column: 30% ethyl acetate in hexane. NMR yield: 25%; isolated yield: 8.3 mg, 15%.

HRMS (DART): *m/z*: $[M+H]^+$, calculated for $C_{15}H_{14}NO_2S^+$: 272.0740; found: 272.0743.

^{1}H NMR ($CDCl_3$, 400 MHz): δ 7.81 (d, *J* = 7.3 Hz, 1H), 7.59 (d, *J* = 7.7 Hz, 1H), 7.31 – 7.26 (m, 3H), 7.18 (t, *J* = 7.2 Hz, 2H), 7.03 (d, *J* = 3.3 Hz, 1H), 6.88 (d, *J* = 8.0 Hz, 2H), 6.51 (d, *J* = 3.7 Hz, 1H), 4.49 (s, 2H)**^{13}C NMR ($CDCl_3$, 101 MHz):** δ 130.6, 129.4, 128.8, 127.1, 127.0, 124.7, 123.4, 121.6, 113.2, 107.8, 59.9.

$\tilde{\nu}_{max}$: 3144, 3113, 3064, 3034, 2924, 1444, 1362, 1259, 1205, 1163, 1122, 1074 cm^{-1}

7a: *N*-methoxy-*N*-methyl-1-phenylmethanesulfonamide

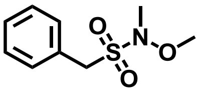

Prepared according to the general procedure described above using *O,N*-Dimethyl-hydroxylamine as the amine. NMR yield: 100%.

HRMS (DART): *m/z*: $[M+NH_4]^+$, calculated for $C_9H_{17}N_2O_3S^+$: 233.0954; found: 233.0956.

^{1}H NMR (CD_3CN, 400 MHz): δ 7.47 – 7.36 (m, 5H), 4.40 (s, 2H), 3.78 (s, 3H), 3.03 (s, 3H).

This product was previously reported in Abdulla *et al*. (2017).[12]

7b: *N*-(benzyloxy)-1-phenylmethanesulfonamide

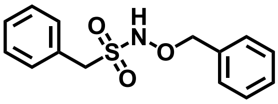

Prepared according to the general procedure described above using *o*-benzylhydroxylamine as the amine. Trace amounts.

HRMS (DART): *m/z*: $[M+NH_4]^+$, calculated for $C_{14}H_{19}N_2O_3S^+$: 295.1111; found: 295.1111.

7c: 1-phenyl-*N*-(piperidin-1-yl)methanesulfonamide

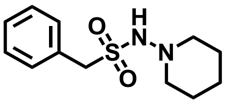

Prepared according to the general procedure described above using 1-aminopiperidine as the amine. Purified by column: 45% ethyl acetate in hexane. White crystals. NMR yield: 100%; isolated yield: 40.6 mg, 78%.

HRMS (DART): *m/z*: $[M+H]^+$, calculated for $C_{12}H_{19}N_2O_2S^+$: 255.1162; found: 255.1161.

^{1}H NMR (CD_3CN, 400 MHz): δ 7.44 – 7.35 (m, 5H), 6.18 (s, 1H), 4.36 (s, 2H), 2.82 (t, *J* = 5.3 Hz, 4H), 1.65 (p, *J* = 5.9 Hz, 4H), 1.39 (p, *J* = 5.9 Hz, 2H). **^{13}C NMR (CD_3CN, 101 MHz):** δ 131.9, 130.6, 129.5, 129.3, 58.8, 56.3, 26.6, 23.9.

$\tilde{\nu}_{max}$: 3178, 3064, 2937, 2920, 2856, 1497, 1454, 1315, 1255, 1149, 1122, 1018 cm^{-1}

7d: *N*'-hexanoyl-1-phenylmethanesulfonohydrazide

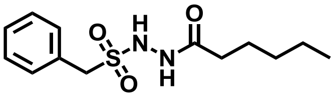

Prepared according to the general procedure described above using hexanehydrazide as the amine. Purified by column: 50% ethyl acetate in hexane. White solid. NMR yield: 31%; NMR yield (IS): 30%; isolated yield: 16.3 mg, 28%.

HRMS (DART): *m/z*: $[M+H]^+$, calculated for $C_{13}H_{21}N_2O_3S^+$: 285.1267; found: 285.1268.

^{1}H NMR (CD_3CN, 400 MHz): δ 8.38 (s, 1H), 7.50 – 7.34 (m, 6H), 4.33 (s, 2H), 2.21 (t, *J* = 6.6 Hz, 2H), 1.63 (p, *J* = 7.5 Hz, 2H), 1.37 – 1.30 (m, 4H), 0.91 (t, *J* = 6.0 Hz, 3H). **^{13}C NMR (CD_3CN, 101 MHz):** δ 173.7, 132.1, 130.1, 129.5, 58.6, 34.3, 32.0, 25.7, 23.0, 14.2.

$\tilde{\nu}_{max}$: 3311, 3132, 3066, 3036, 2953, 2928, 2870, 1687, 1591, 1518, 1497, 1456, 1335, 1153 cm^{-1}

NMR spectra of novel compounds

3b: *N*-(*sec*-butyl)-1-phenylmethanesulfonamide

^{1}H spectrum (CD_3CN, 400 MHz):

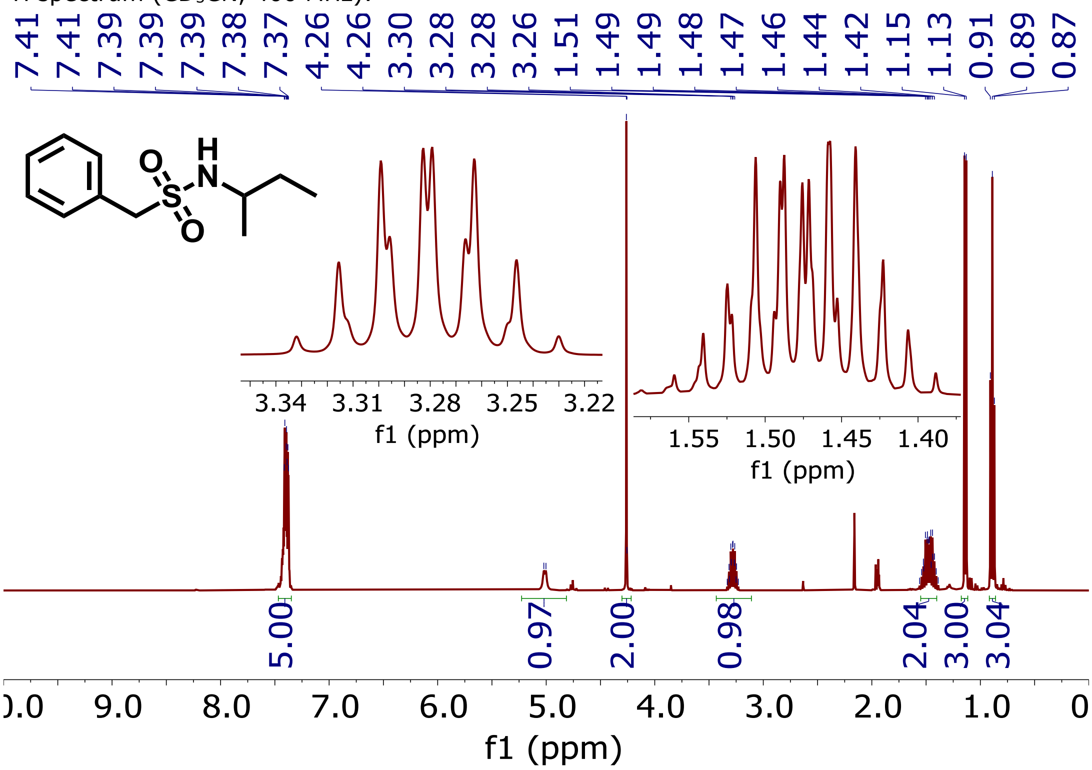

^{13}C spectrum (CD_3CN, 101 MHz):

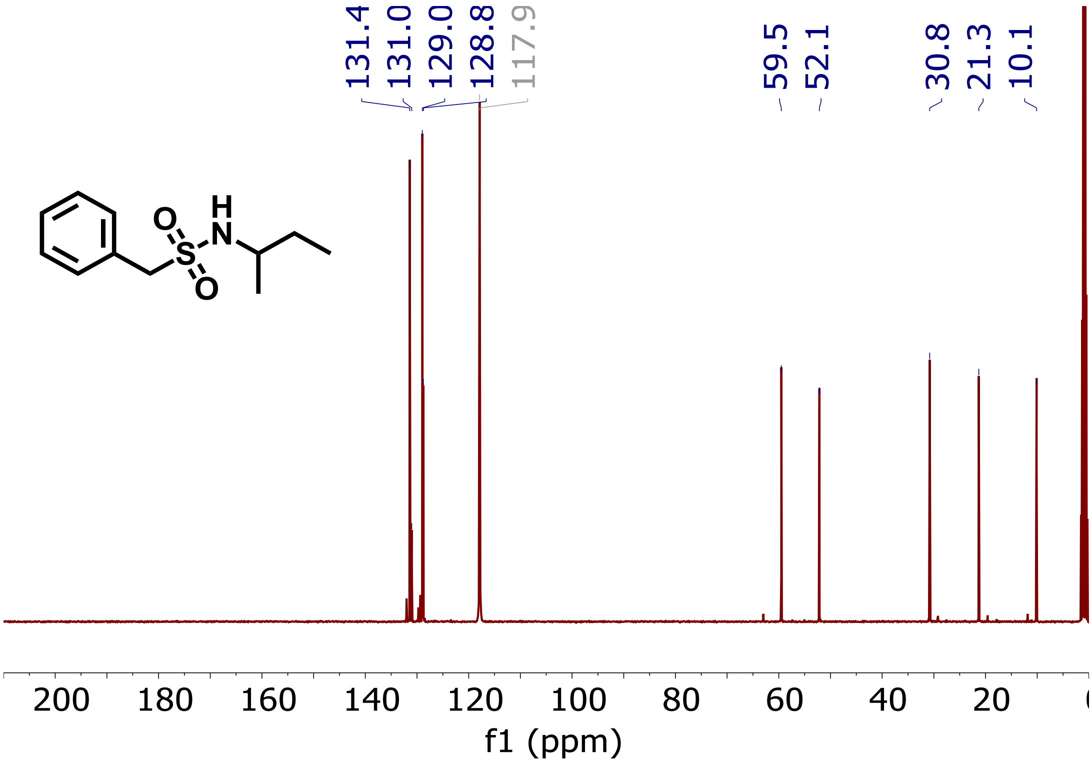

3d: *N*-butyl-*N*-methyl-1-phenylmethanesulfonamide

^{1}H spectrum (CD_3CN, 400 MHz):

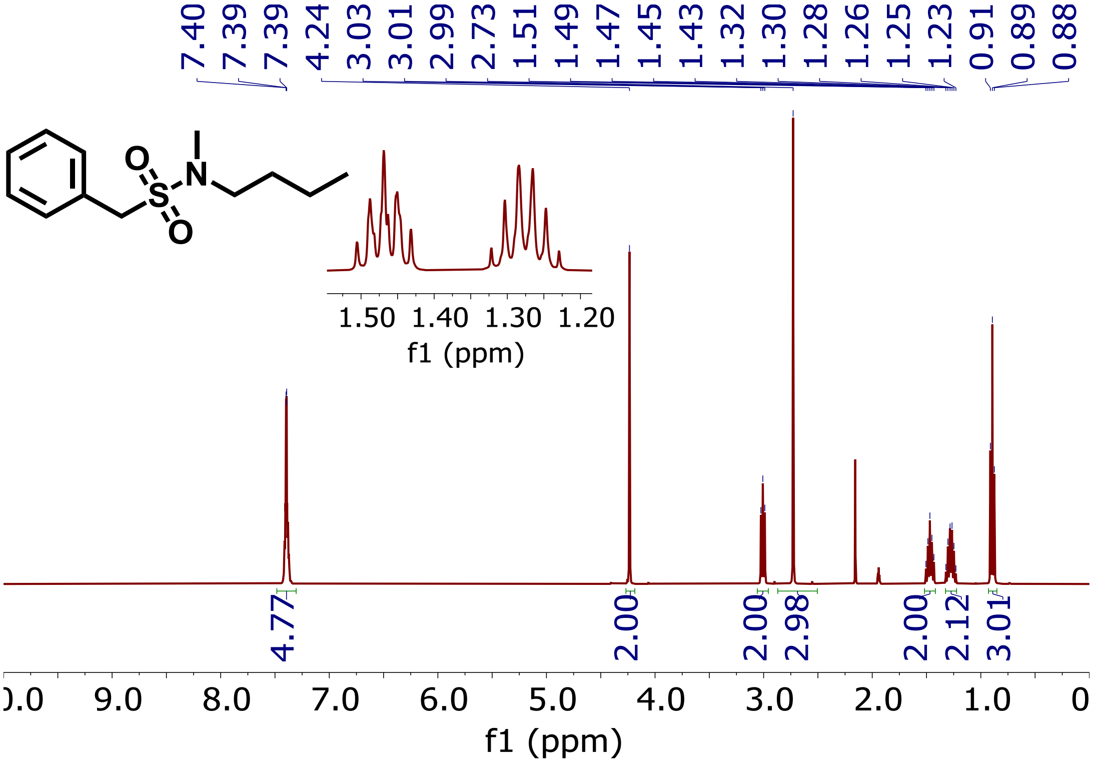

^{13}C spectrum (CD_3CN, 101 MHz):

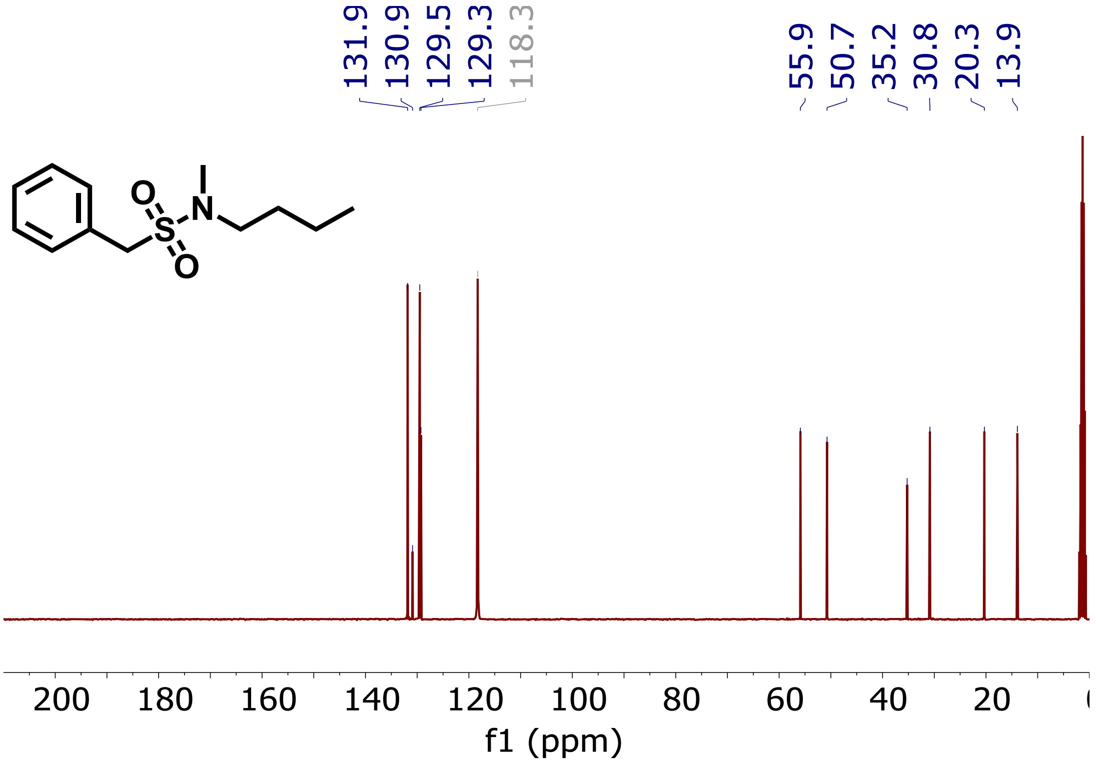

3e: *N*-butyl-*N*-ethyl-1-phenylmethanesulfonamide

^{1}H spectrum (CD_3CN, 400 MHz):

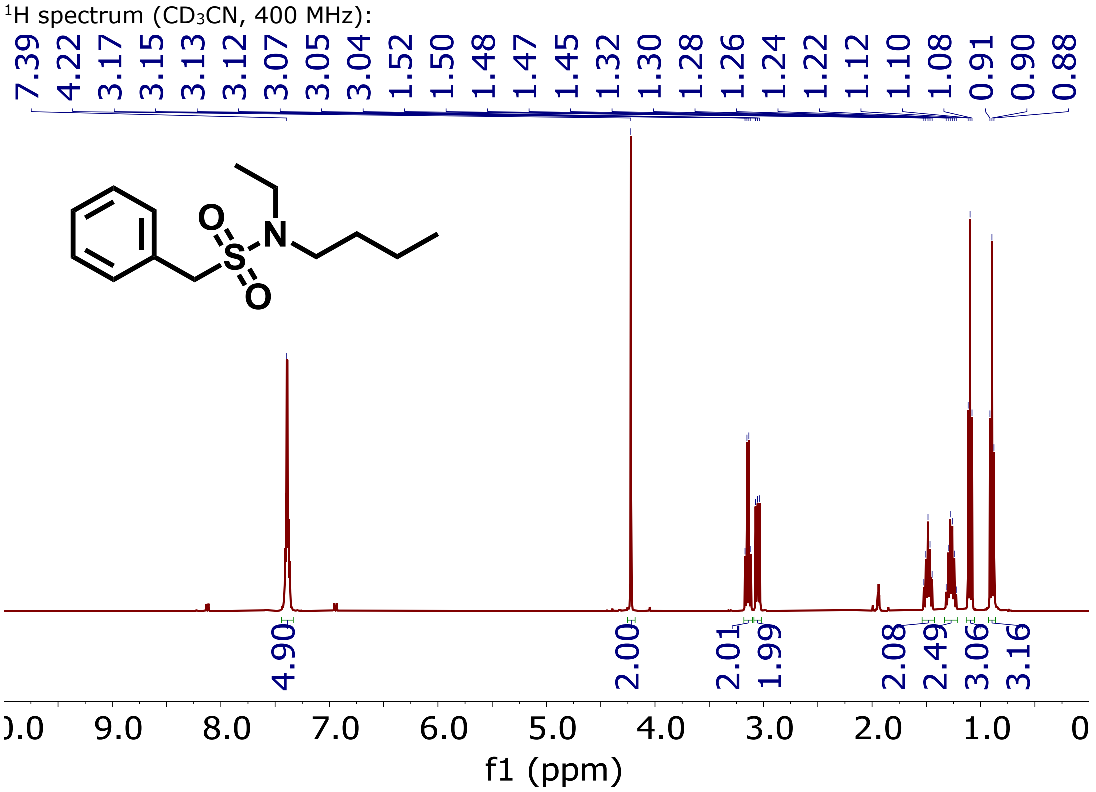

^{13}C spectrum (CD_3CN, 101 MHz):

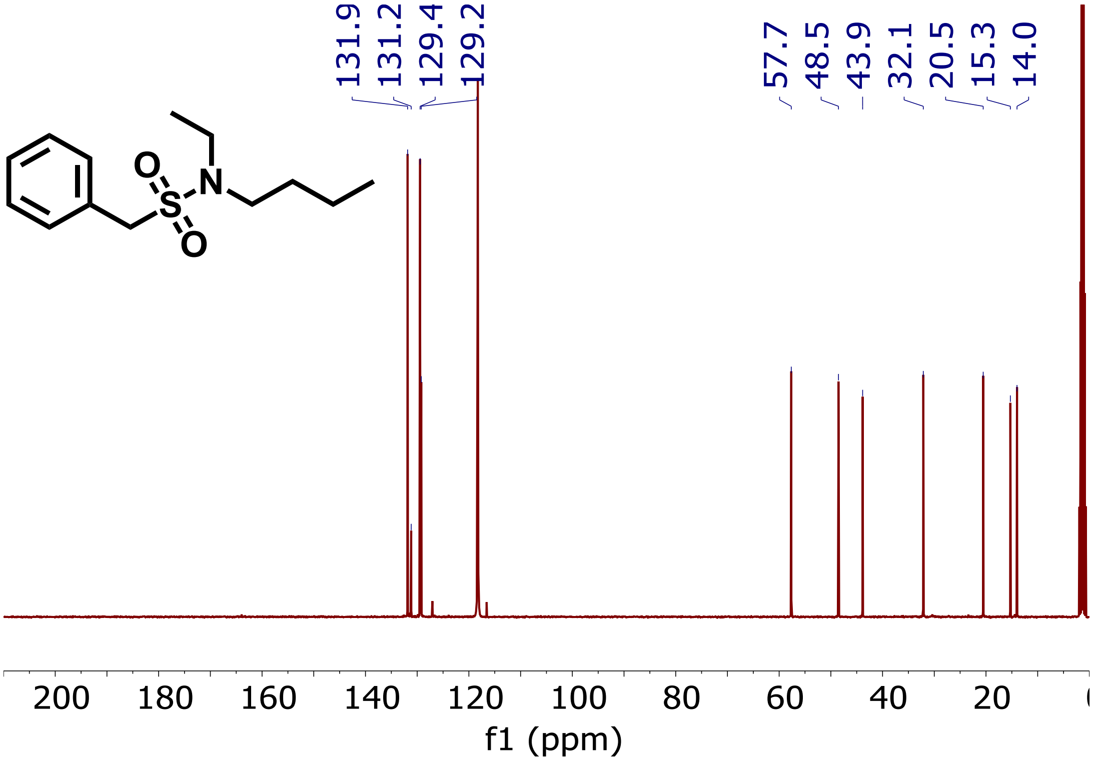

3h: 1-(benzylsulfonyl)azetidine

^{1}H spectrum ($CDCl_3$, 400 MHz):

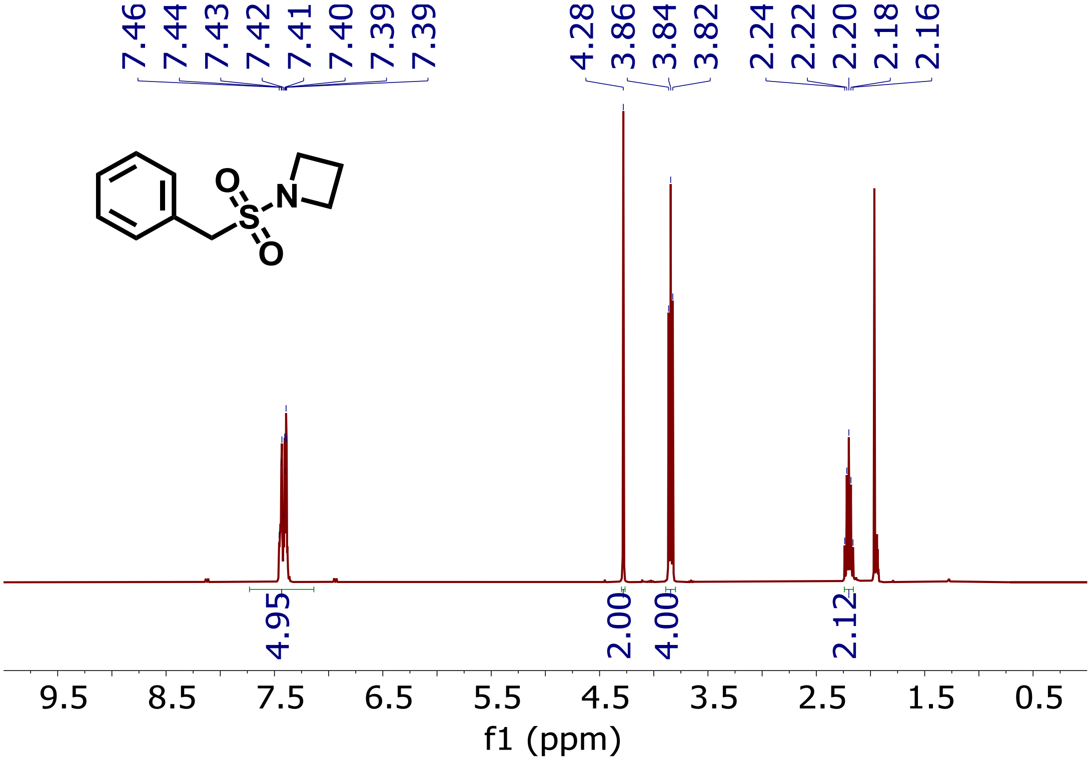

^{13}C spectrum ($CDCl_3$, 101 MHz):

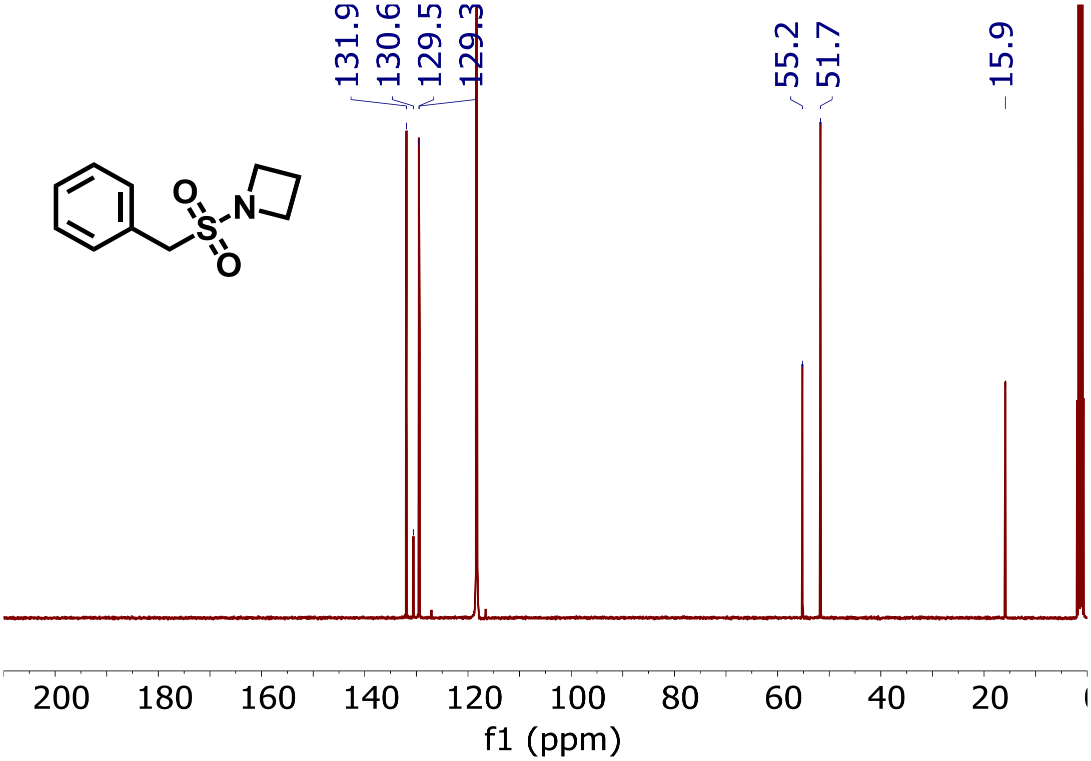

3k: 1-(benzylsulfonyl)azepane

^{1}H spectrum ($CDCl_3$, 400 MHz):

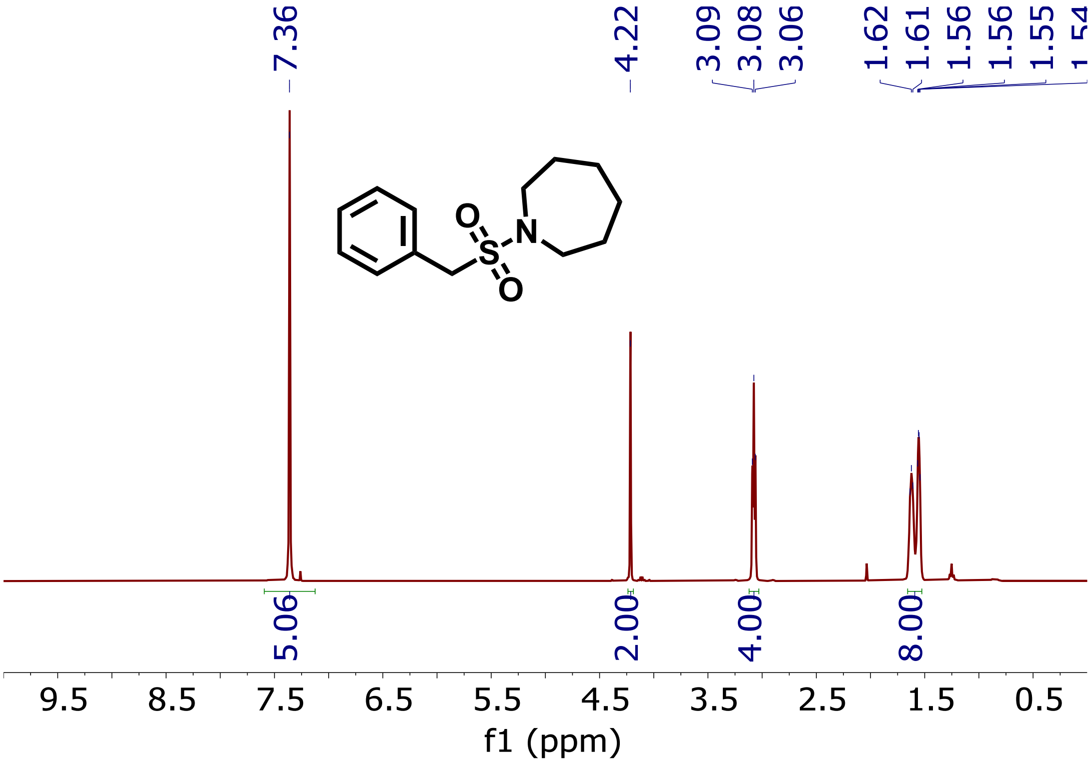

^{13}C spectrum ($CDCl_3$, 101 MHz):

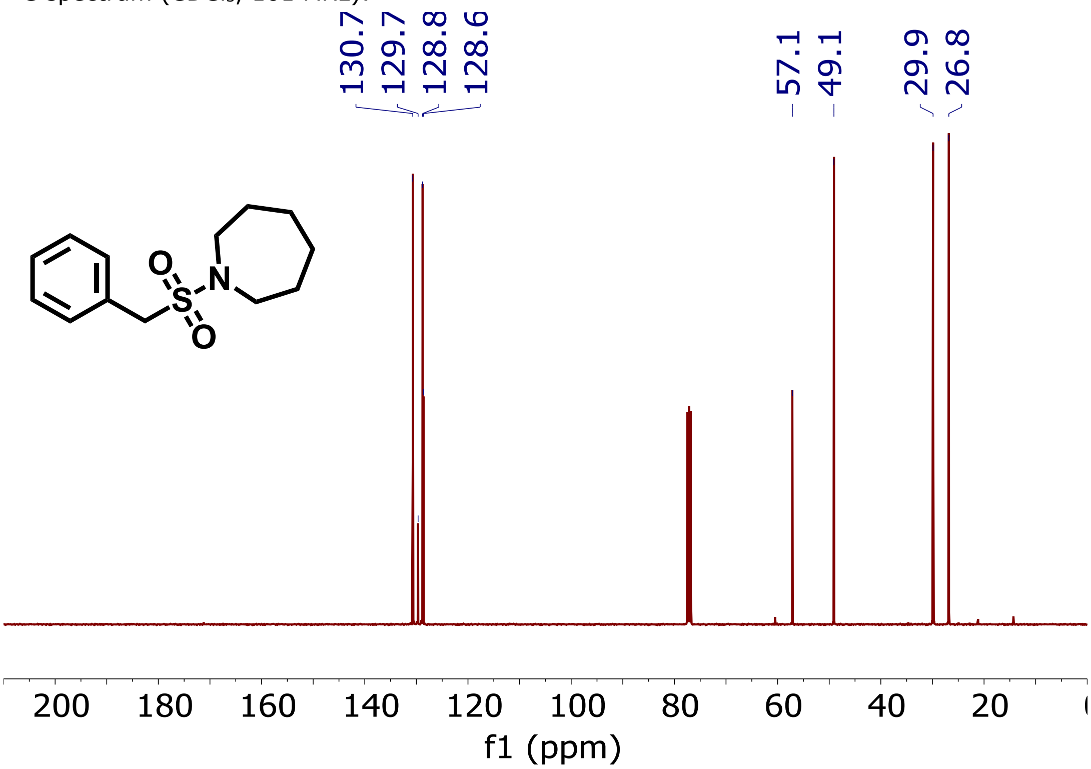

3l: 1-(benzylsulfonyl)azocane

^{1}H spectrum (CD_3CN, 400 MHz):

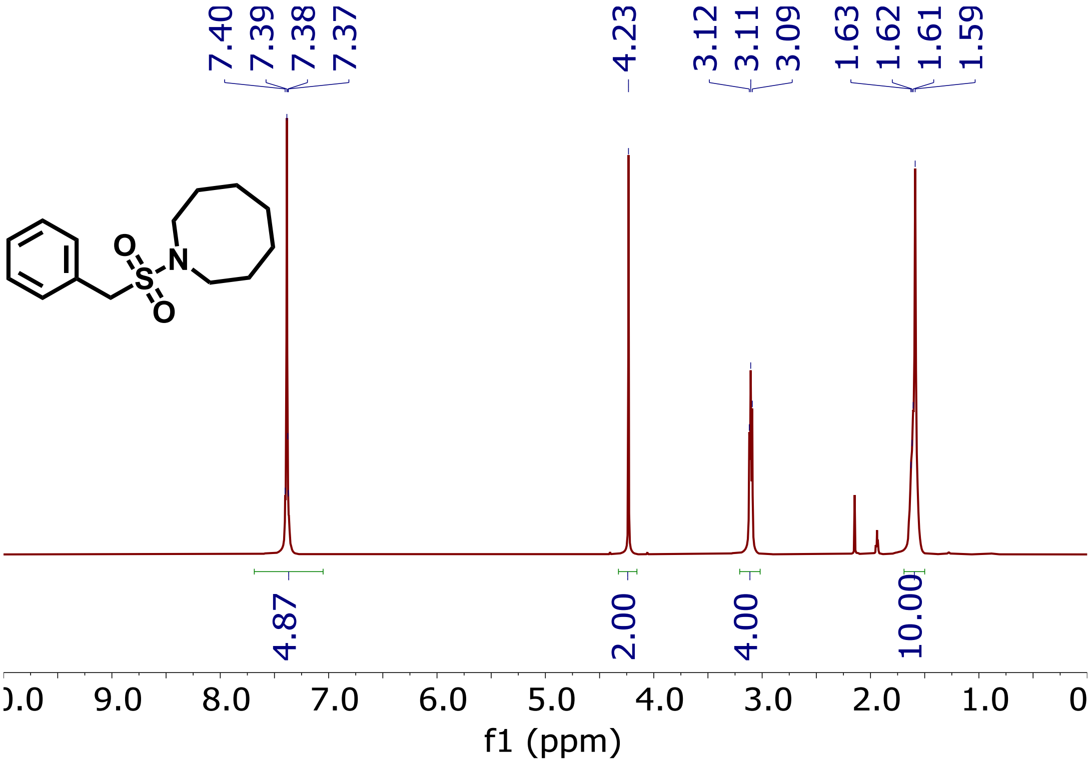

^{13}C spectrum (CD_3CN, 101 MHz):

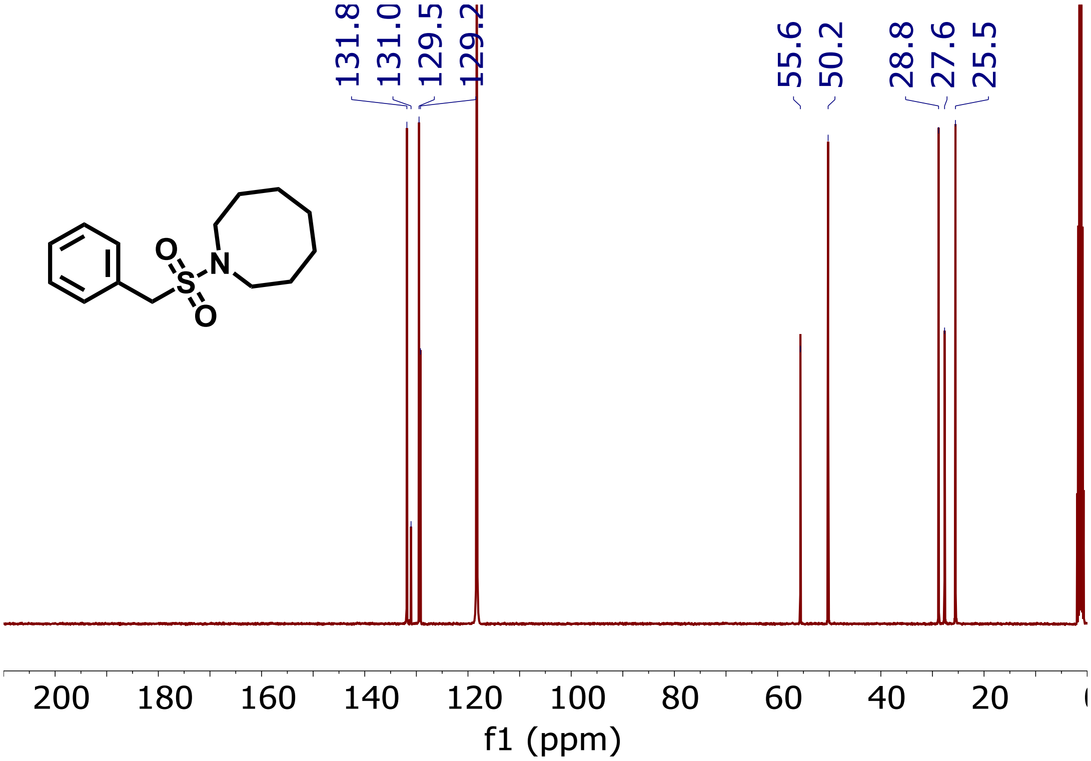

<u>3m: benzyl (benzylsulfonyl)glycinate</u>

^{1}H spectrum (CD_3CN, 400 MHz):

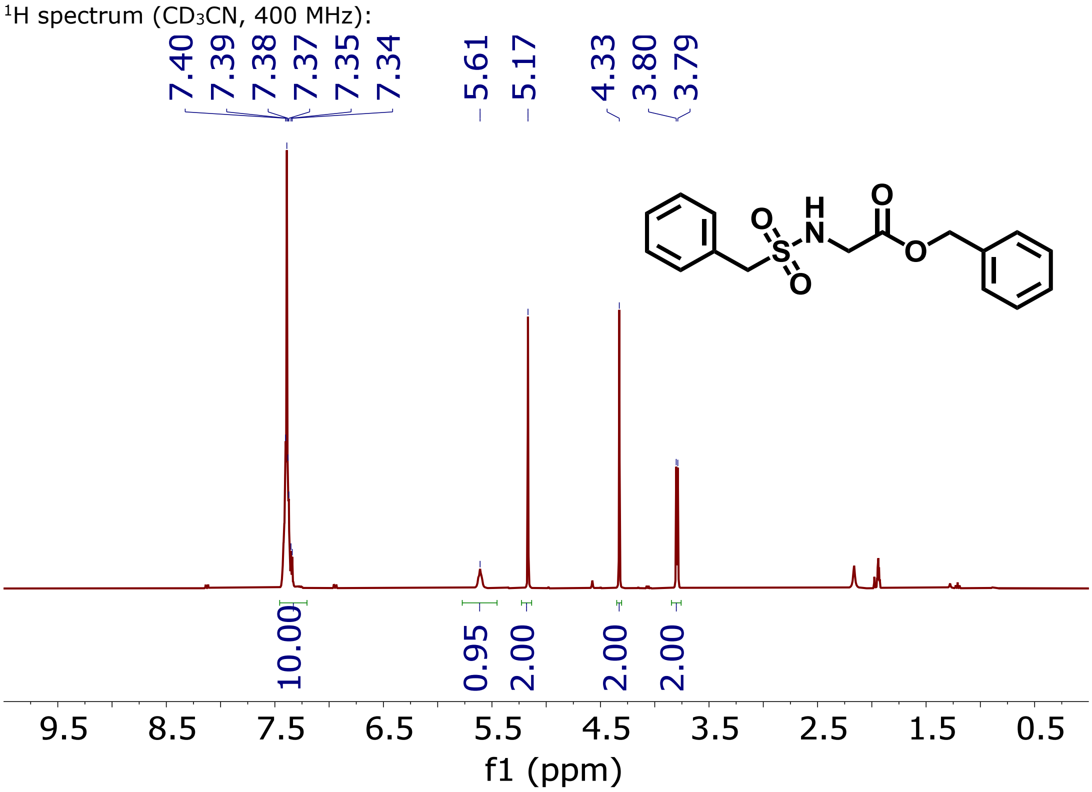

^{13}C spectrum (CD_3CN, 101 MHz):

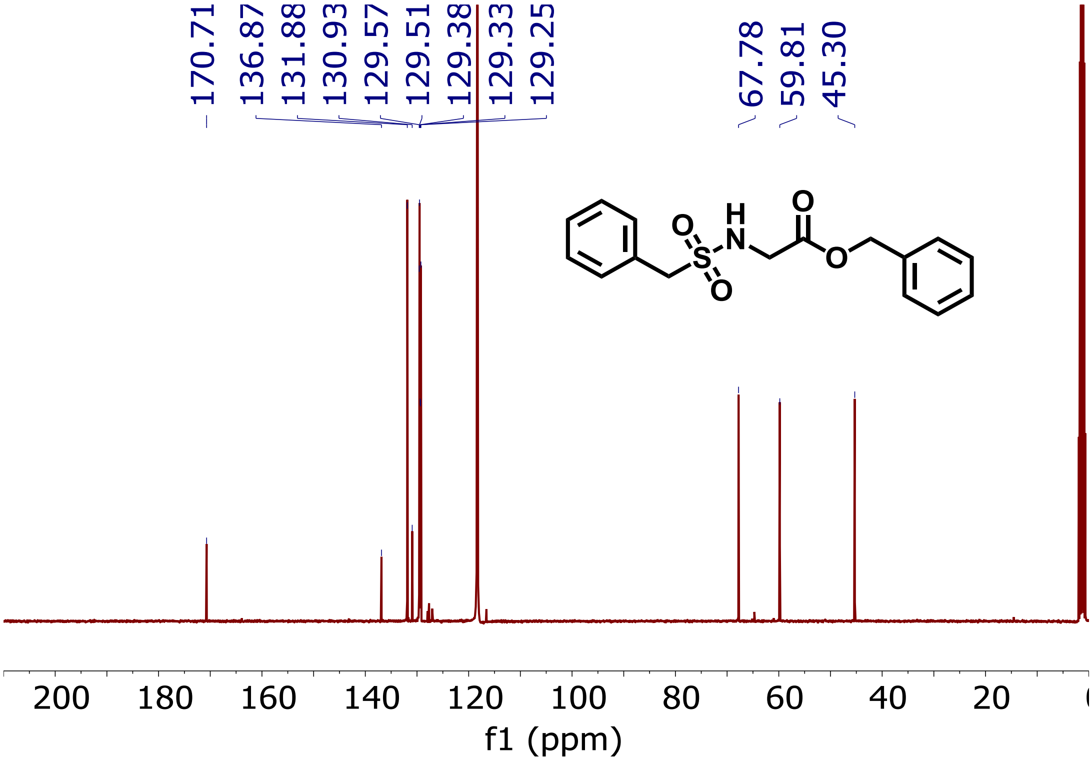

3n: benzyl (benzylsulfonyl)-*L*-prolinate

^{1}H spectrum (CD_3CN, 400 MHz):

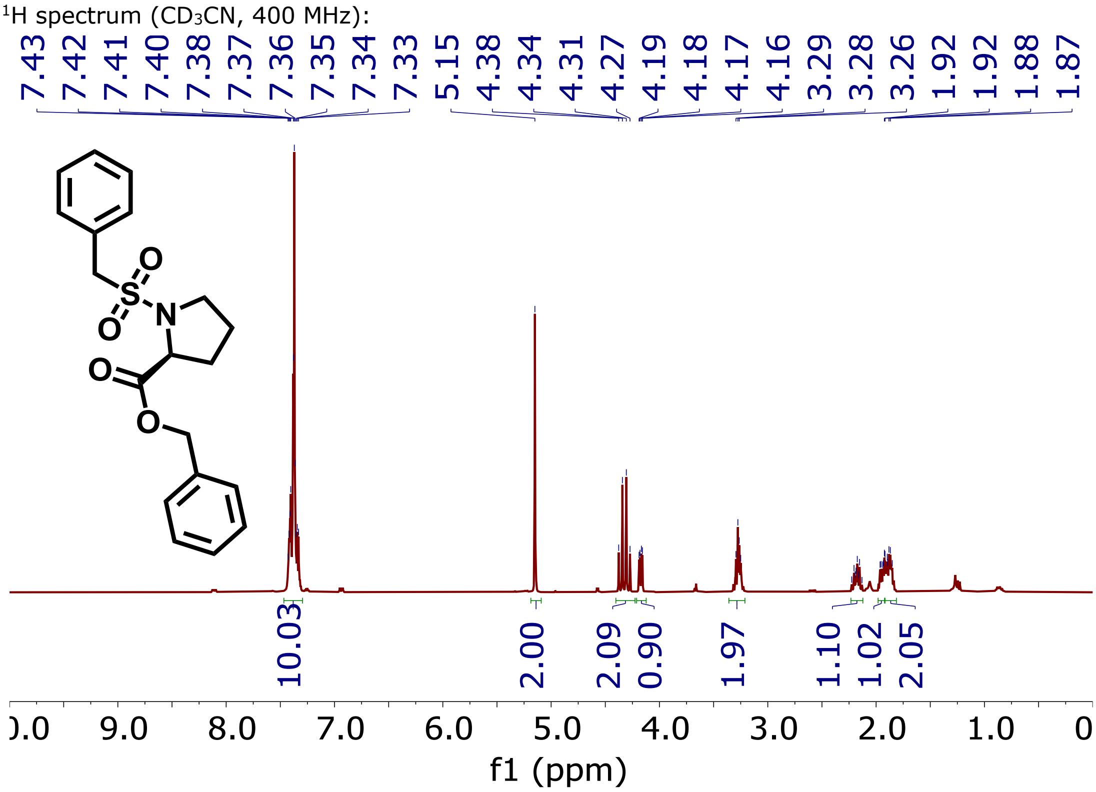

^{13}C spectrum (CD_3CN, 101 MHz):

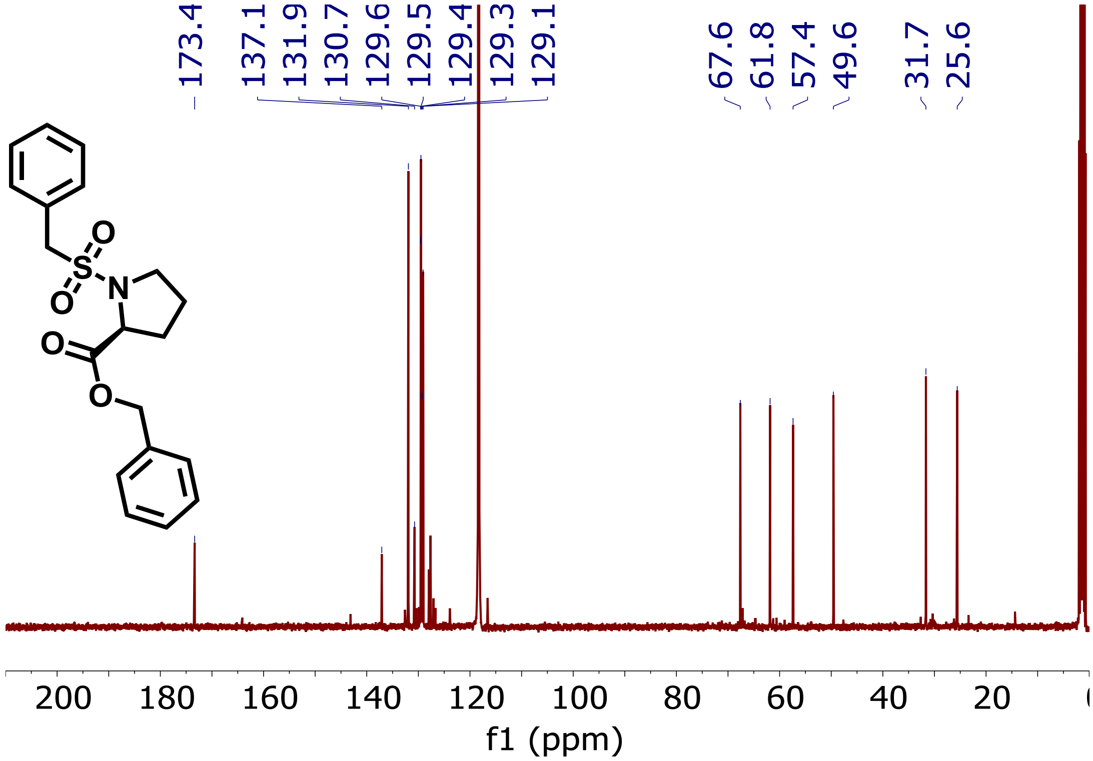

3o: benzyl (benzylsulfonyl)-*L*-tryptophanate

^{1}H spectrum (CD_3CN, 400 MHz):

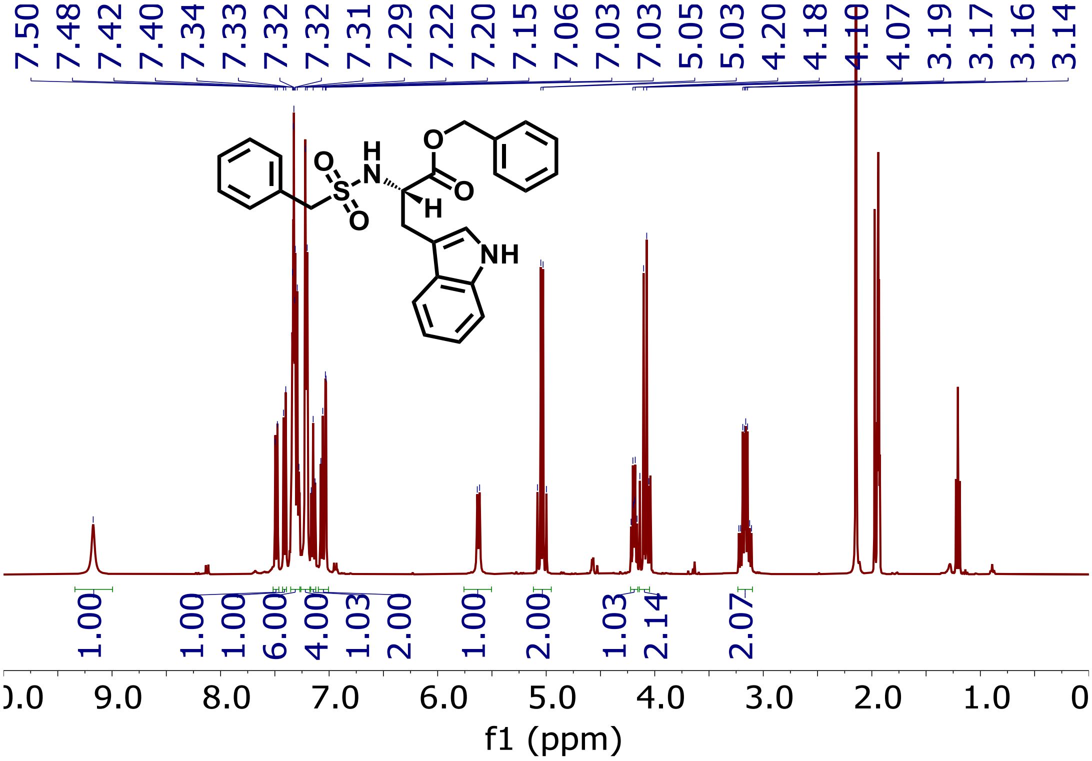

Zoomed-in sections:

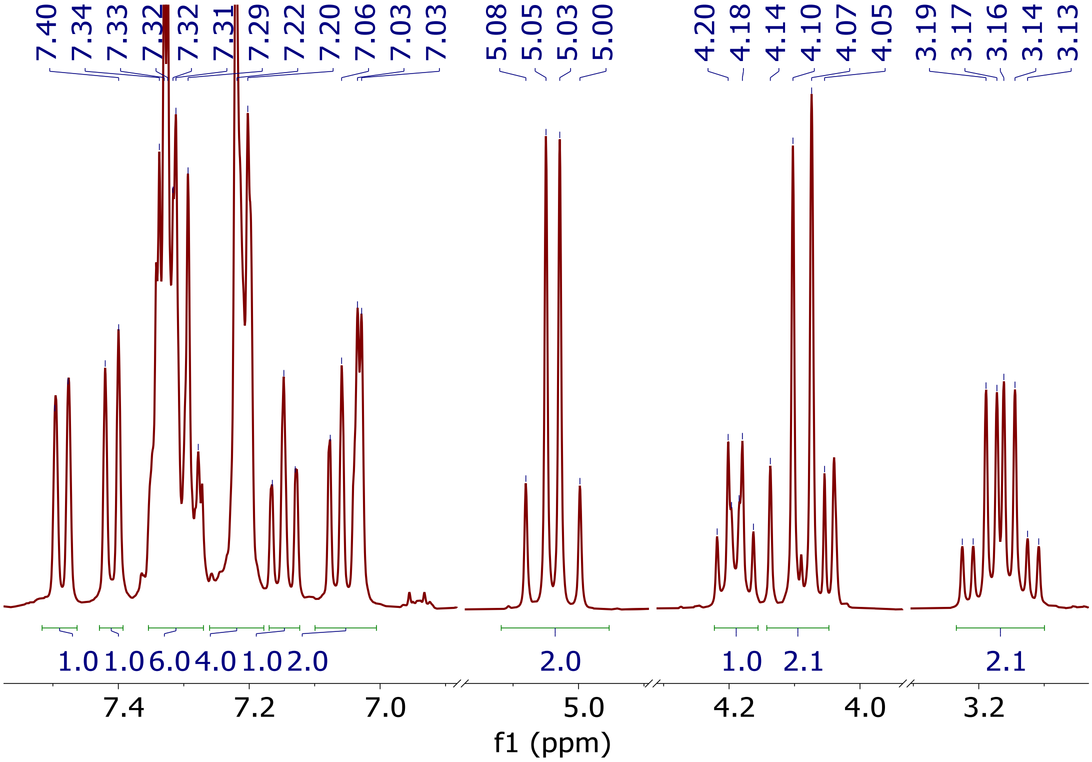

^{13}C spectrum (CD_3CN, 101 MHz):

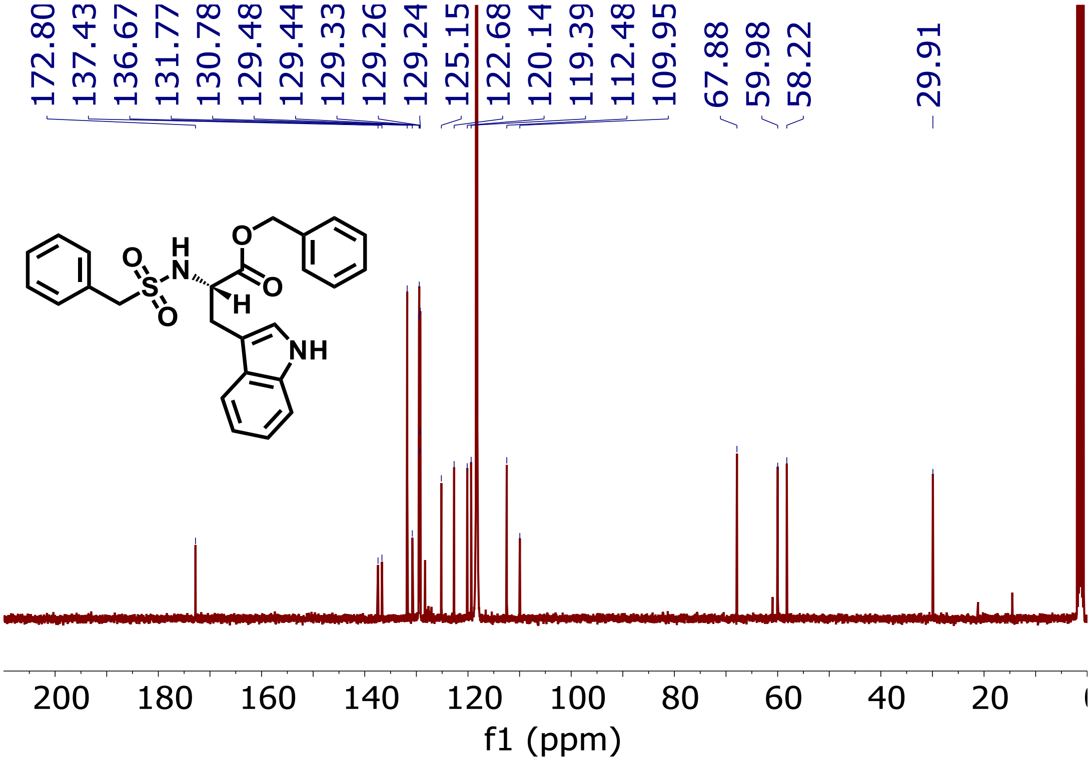

5b: *N*-methyl-1-phenyl-*N*-(*p*-tolyl)methanesulfonamide

^{1}H spectrum (CD_3CN, 400 MHz):

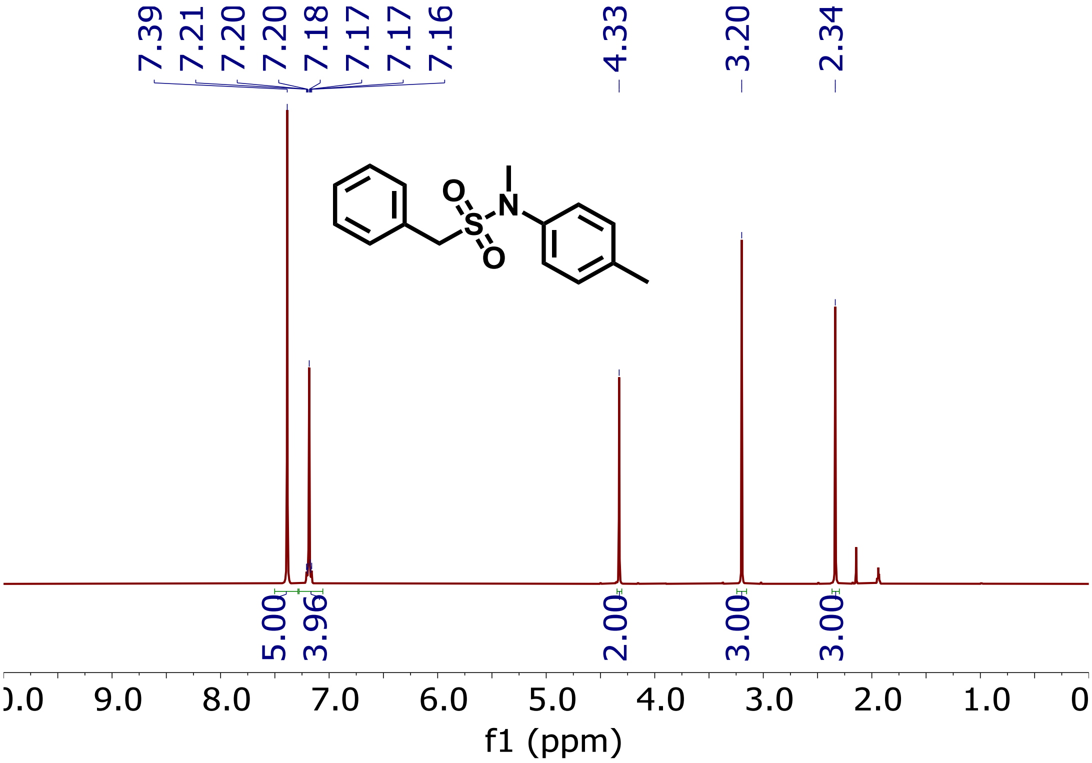

^{13}C spectrum (CD_3CN, 101 MHz):

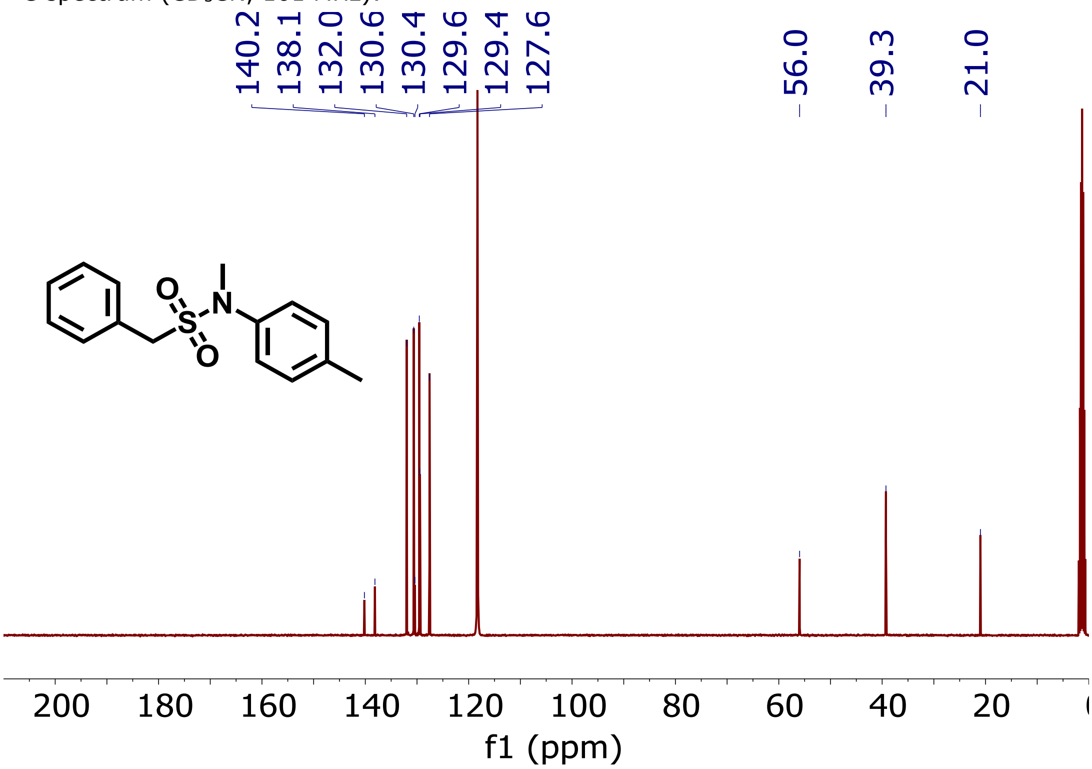

5c: *N*-(4-fluorophenyl)-*N*-methyl-1-phenylmethanesulfonamide

^{1}H spectrum (CD_3CN, 400 MHz):

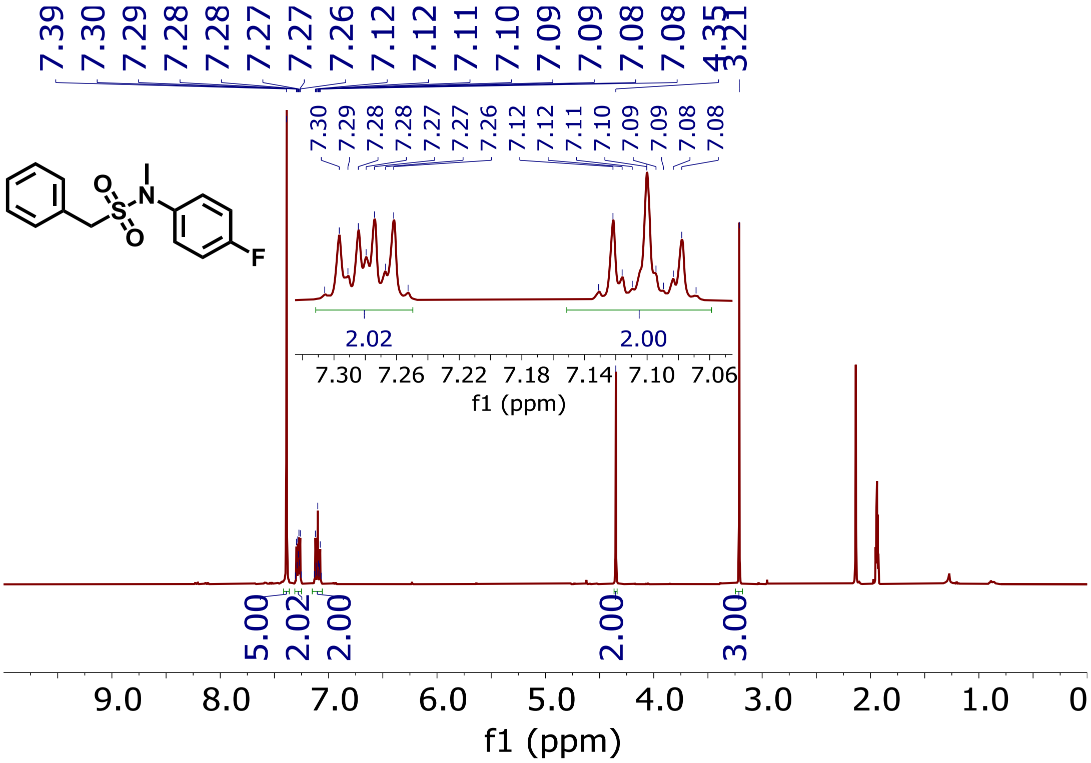

^{13}C spectrum (CD_3CN, 101 MHz):

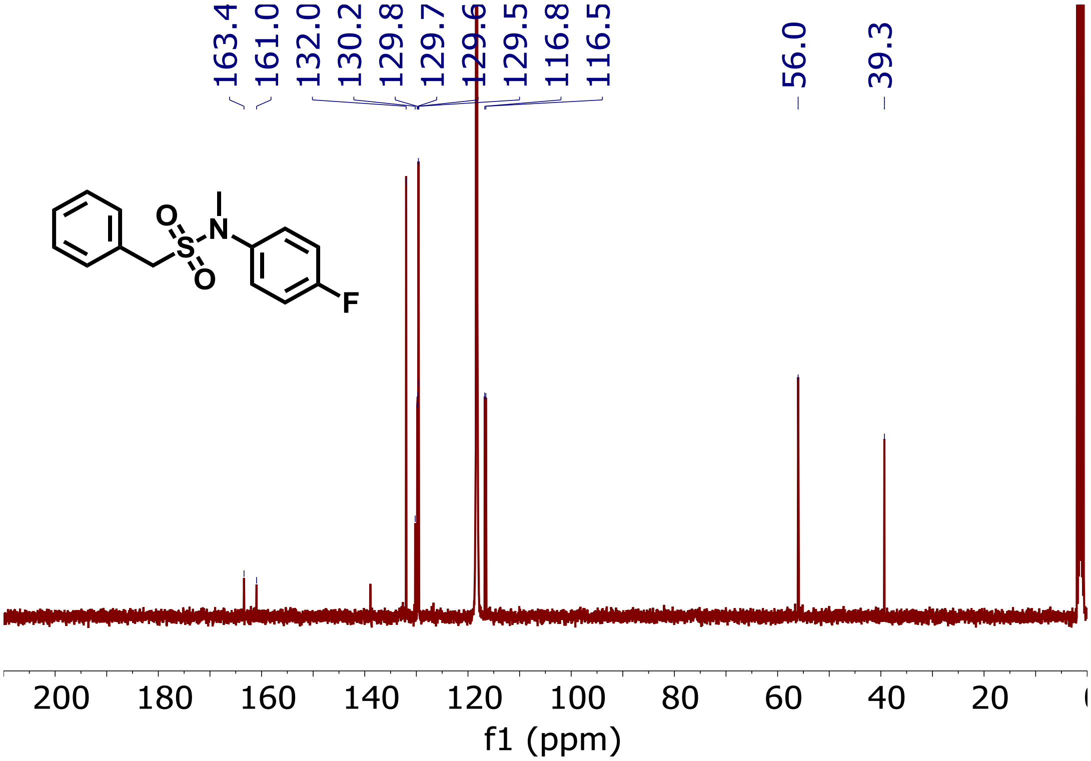

^{19}F spectrum (CD_3CN, 376 MHz). Spectrum with proton decoupling shown in large, zoom-in of a spectrum without decoupling shown in insert:

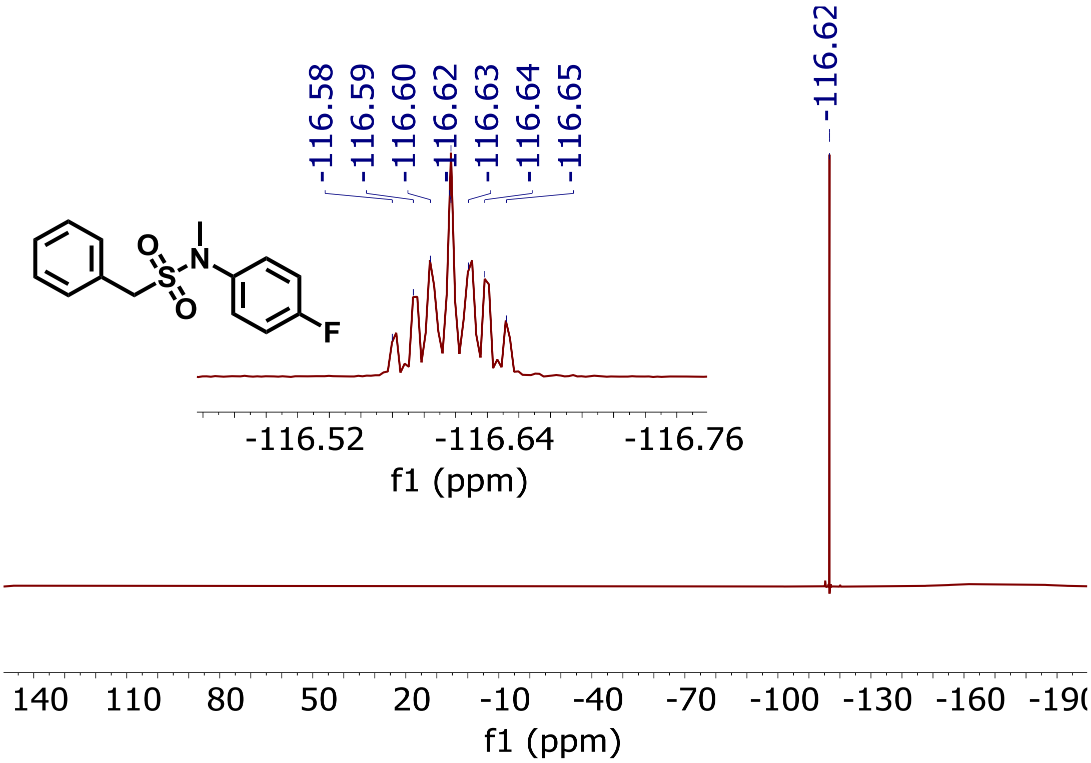

5d: *N*-(4-chlorophenyl)-*N*-methyl-1-phenylmethanesulfonamide

^{1}H spectrum (CD_3CN, 400 MHz):

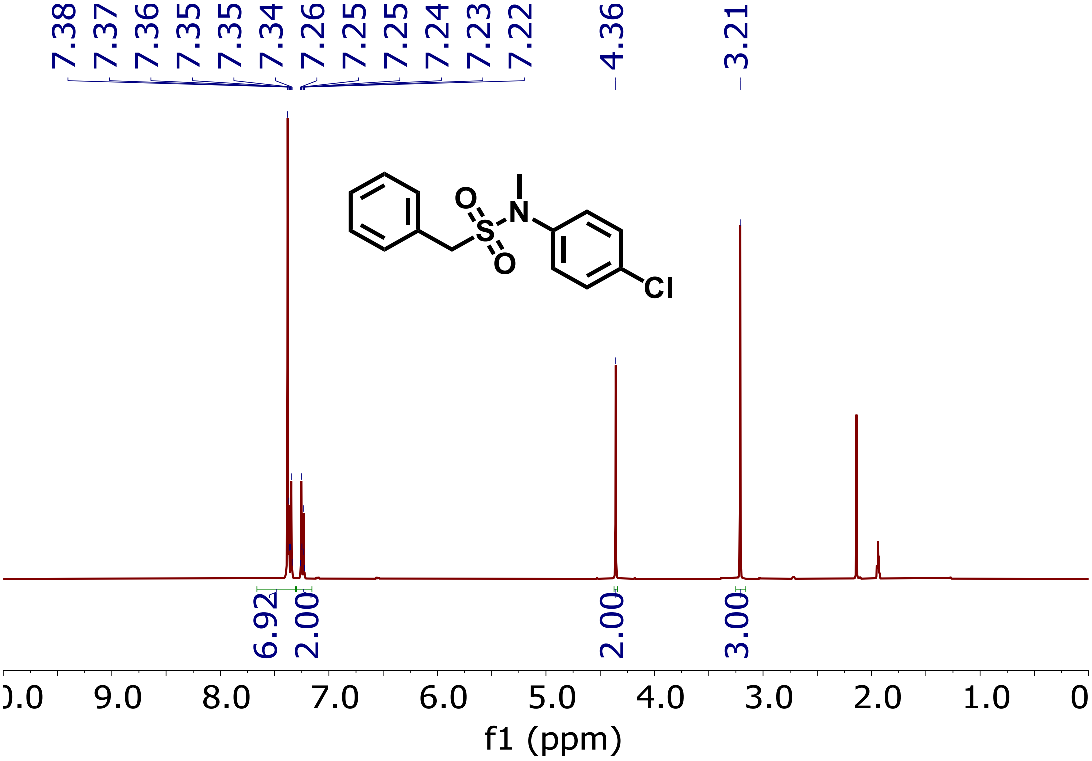

^{13}C spectrum (CD_3CN, 101 MHz):

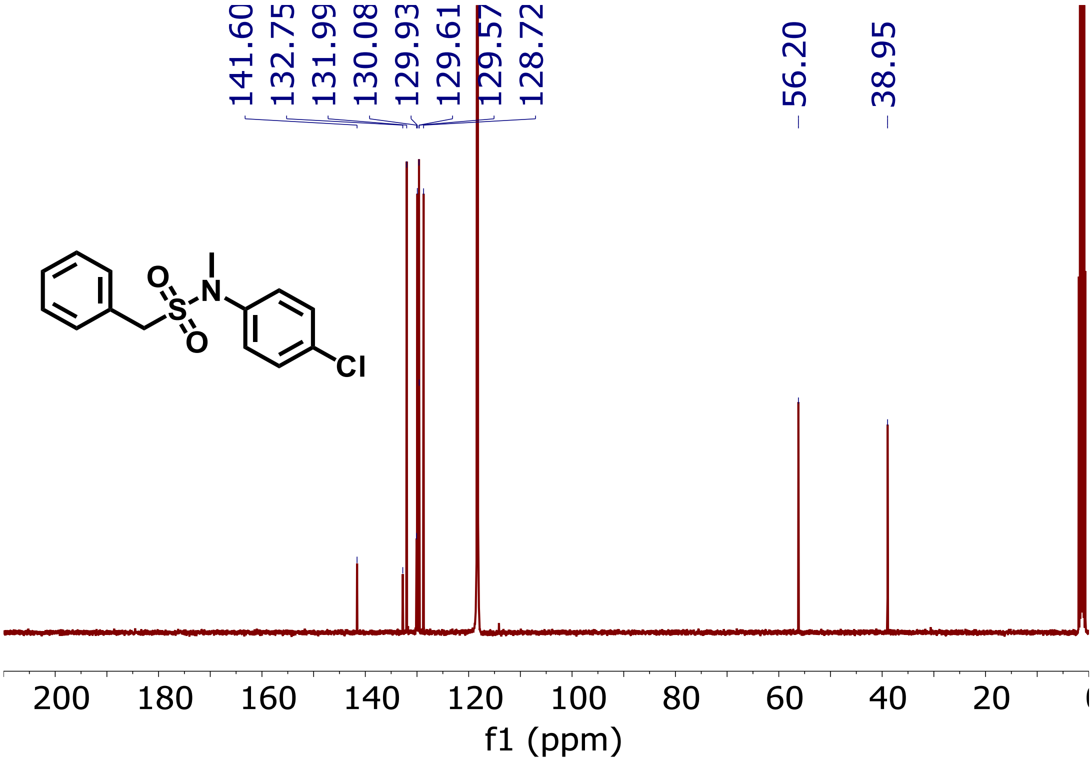

5e: methyl 4-((*N*-methyl-1-phenylmethyl)sulfonamido)benzoate

^{1}H spectrum (CD_3CN, 400 MHz):

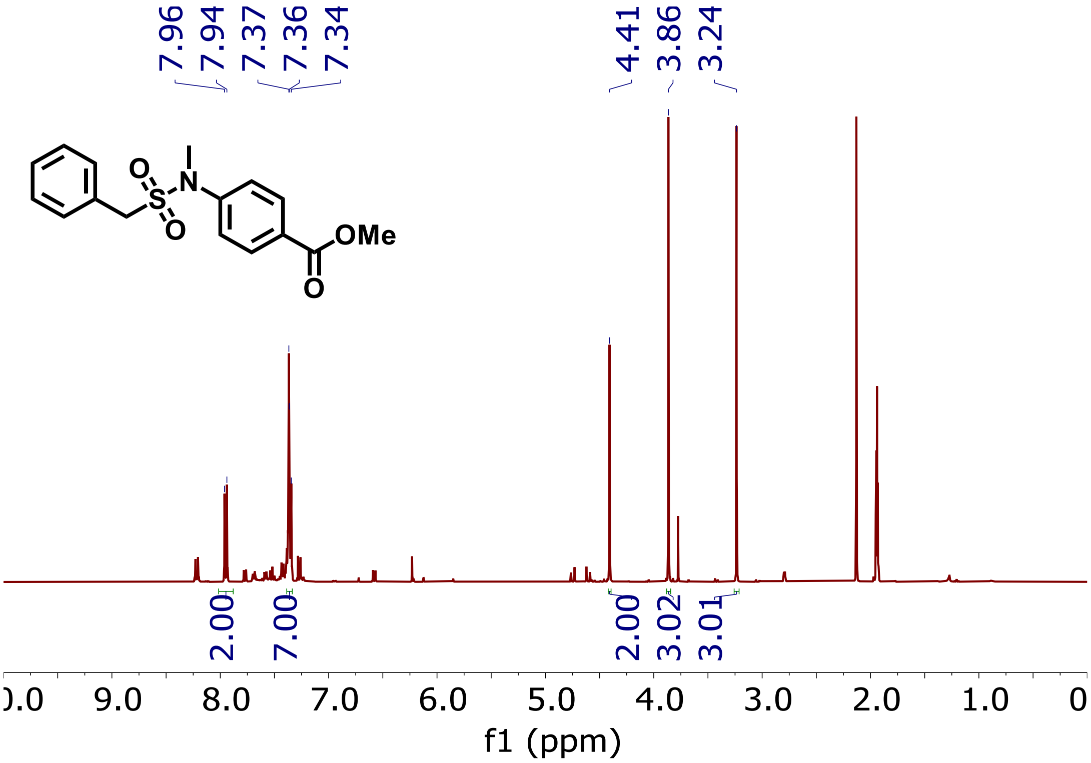

^{13}C spectrum (CD_3CN, 101 MHz):

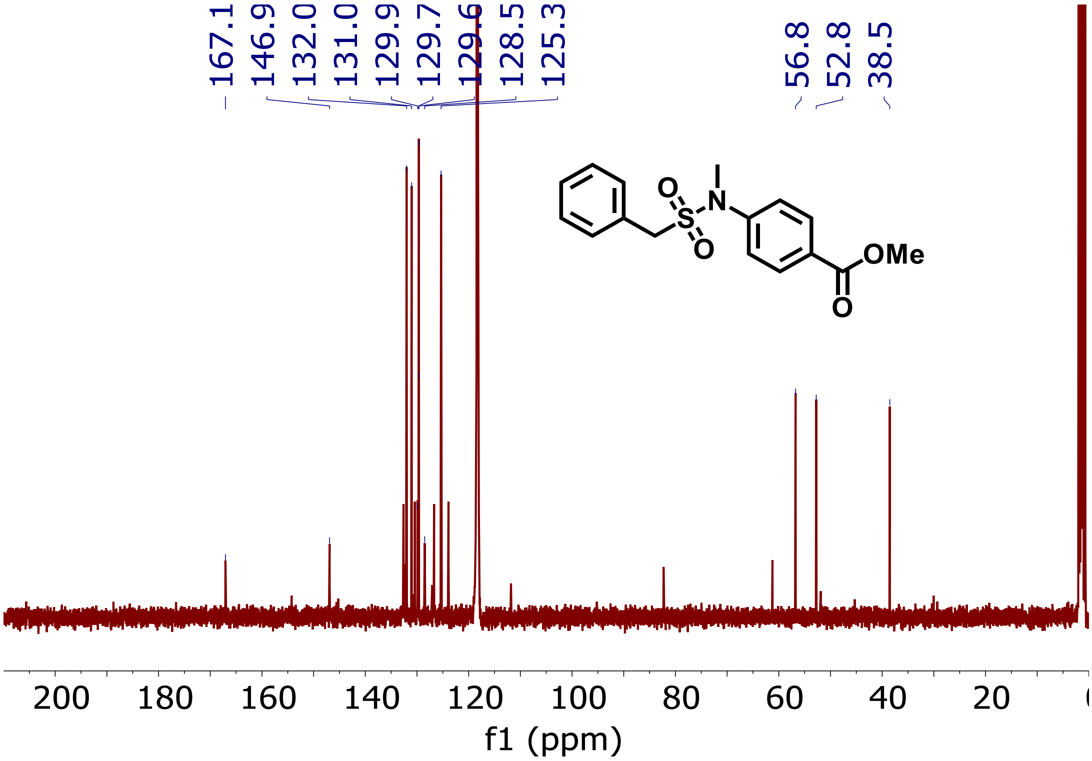

5g: *N*-(4-aminophenyl)-*N*-methyl-1-phenylmethanesulfonamide

^{1}H spectrum (CD_3CN, 400 MHz):

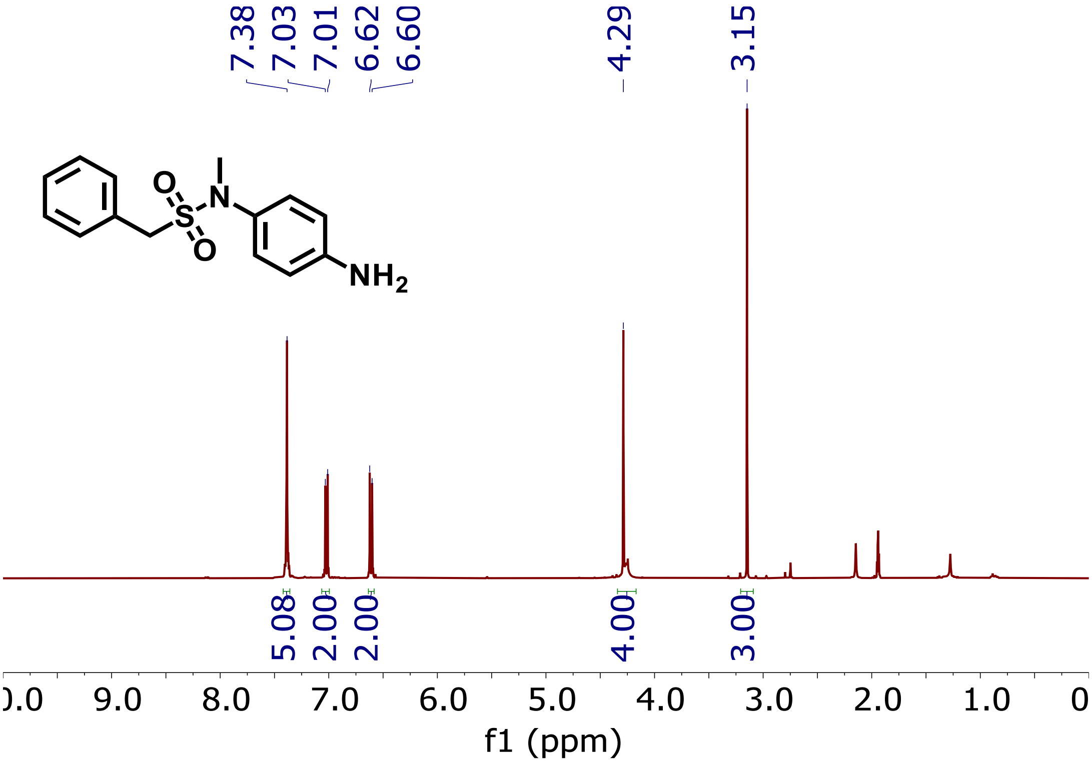

^{13}C spectrum (CD_3CN, 101 MHz):

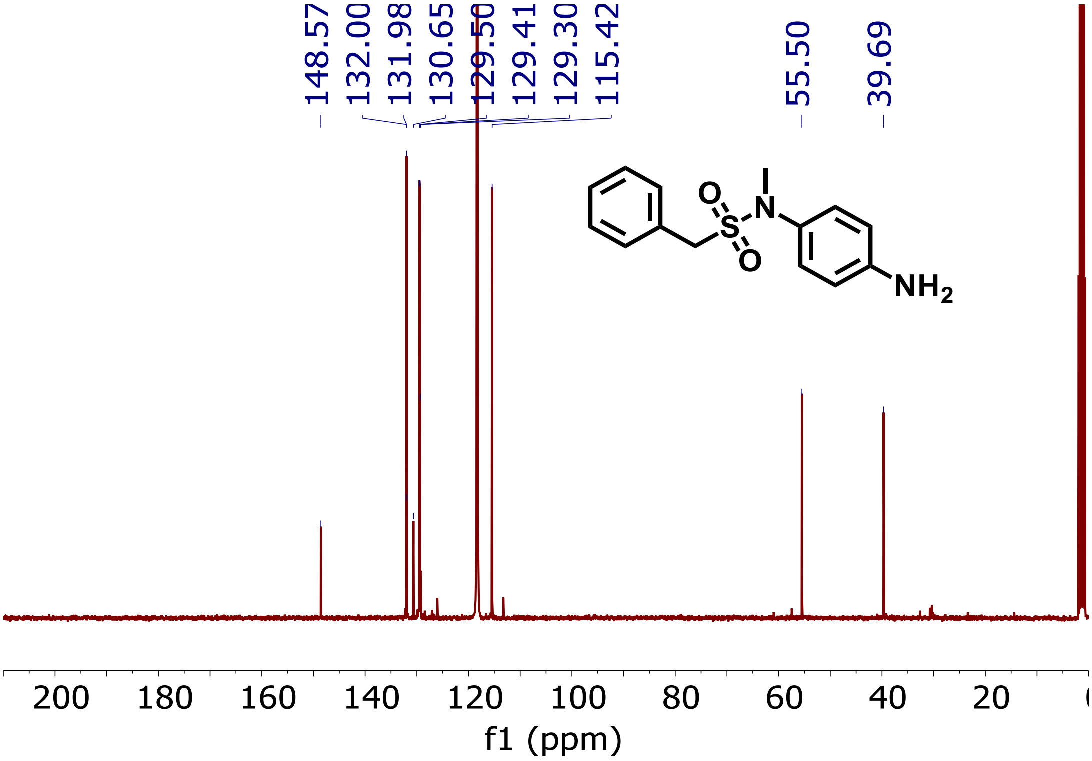

5j: *N*-(4-fluorophenyl)-1-phenylmethanesulfonamide

^{1}H spectrum (CD_3CN, 400 MHz):

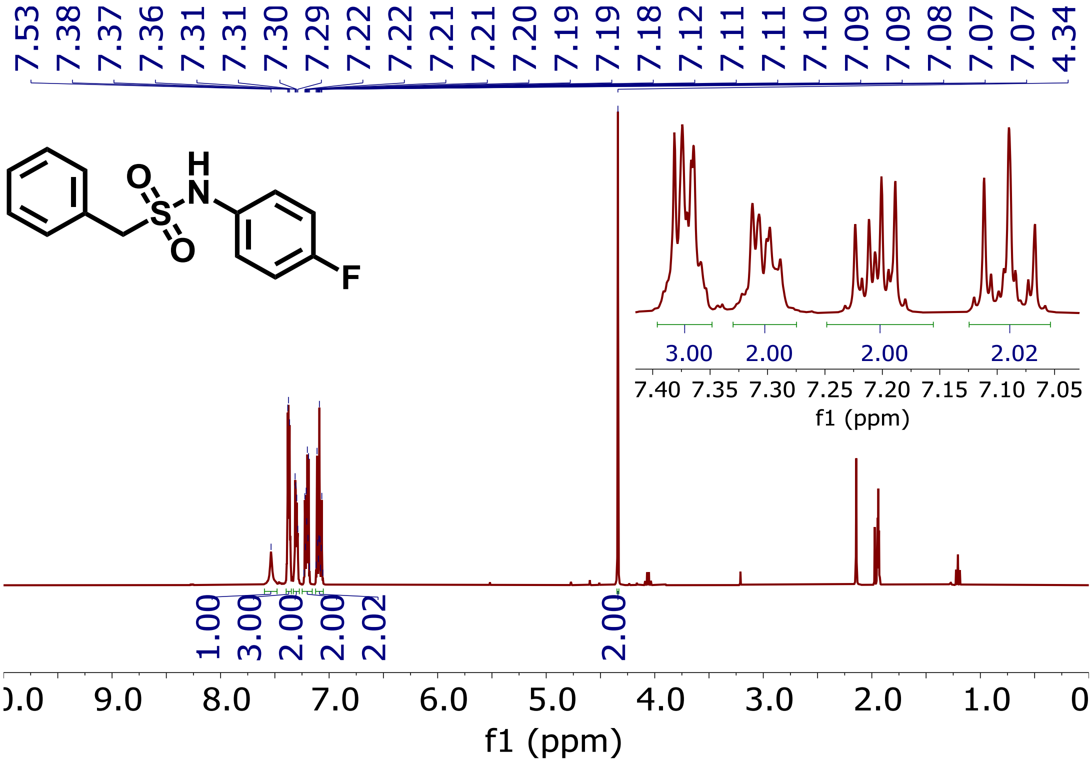

^{13}C spectrum (CD_3CN, 101 MHz):

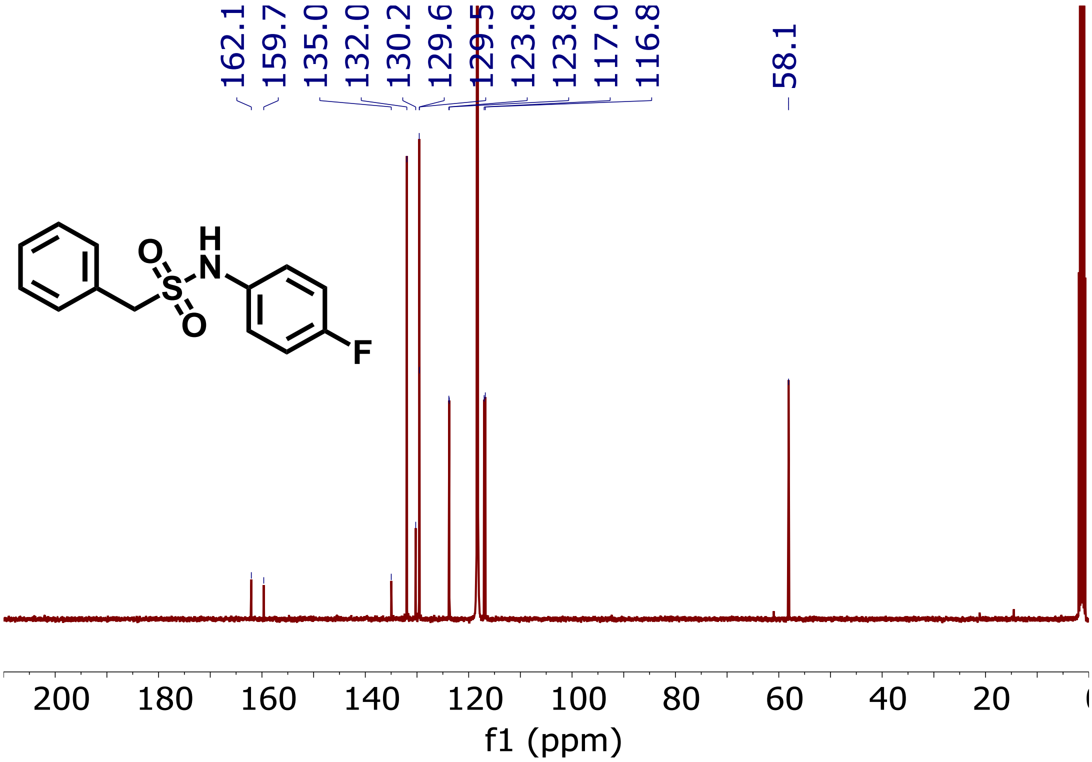

^{19}F spectrum (CD_3CN, 376 MHz):

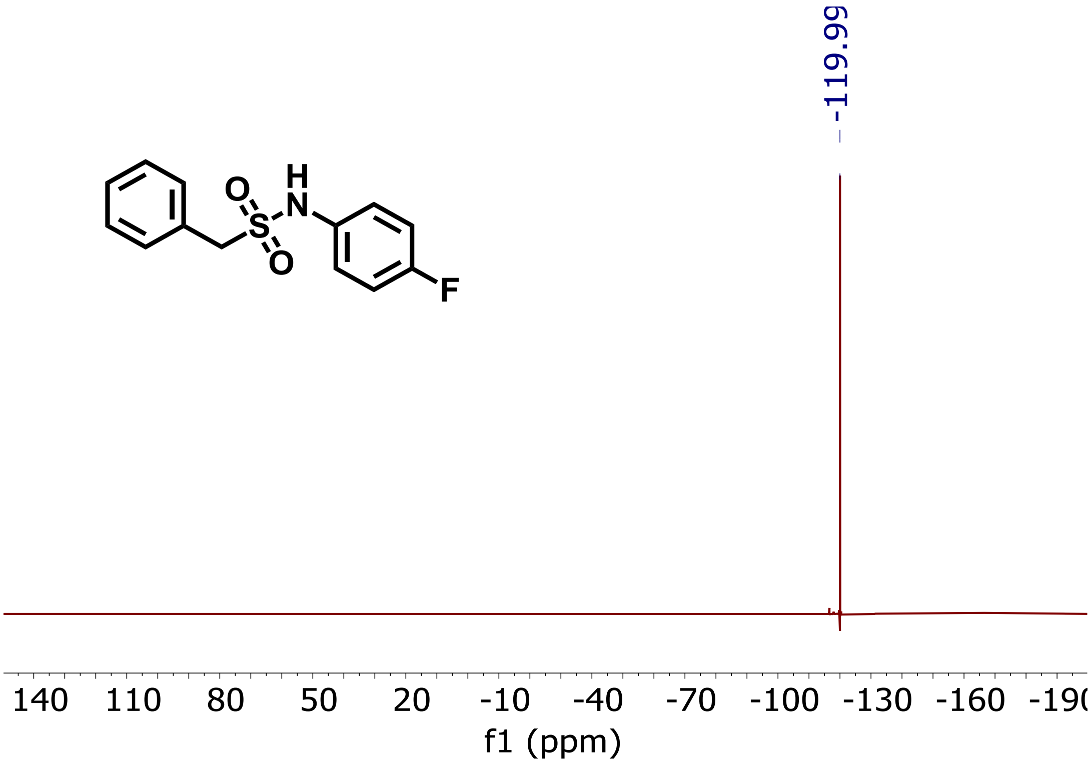

5o: *N*-(3-acetylphenyl)-1-phenylmethanesulfonamide

^{1}H spectrum (CD_3CN, 400 MHz):

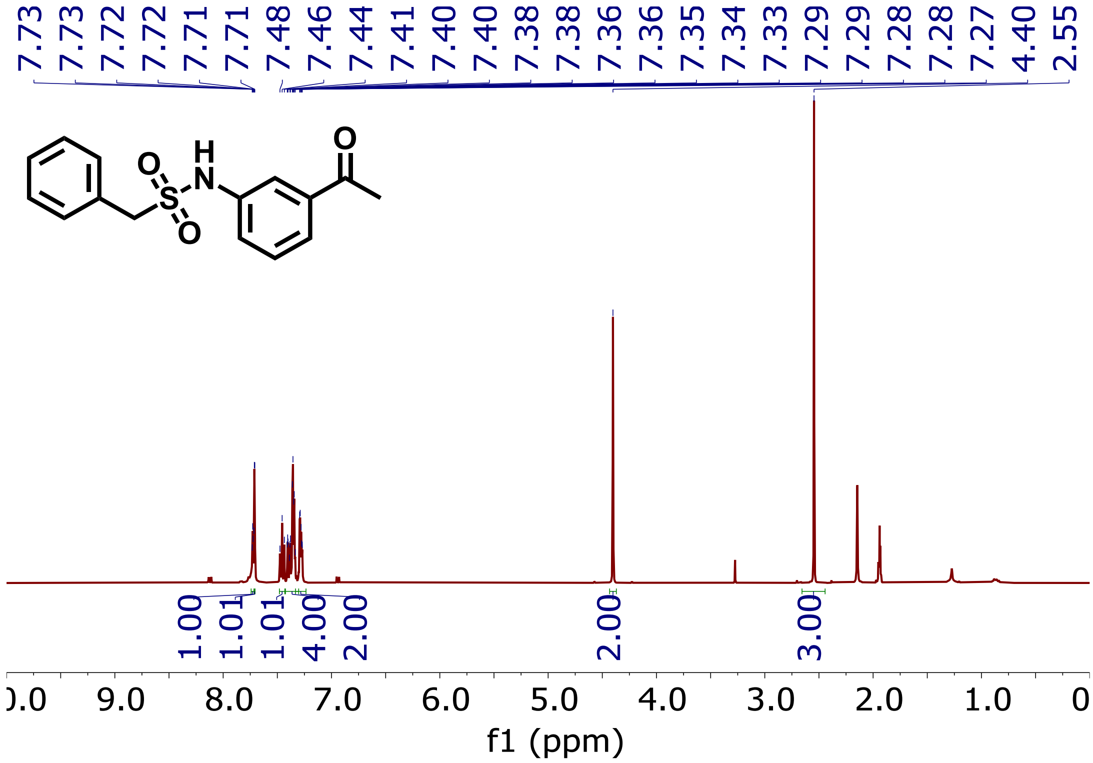

^{13}C spectrum (CD_3CN, 101 MHz):

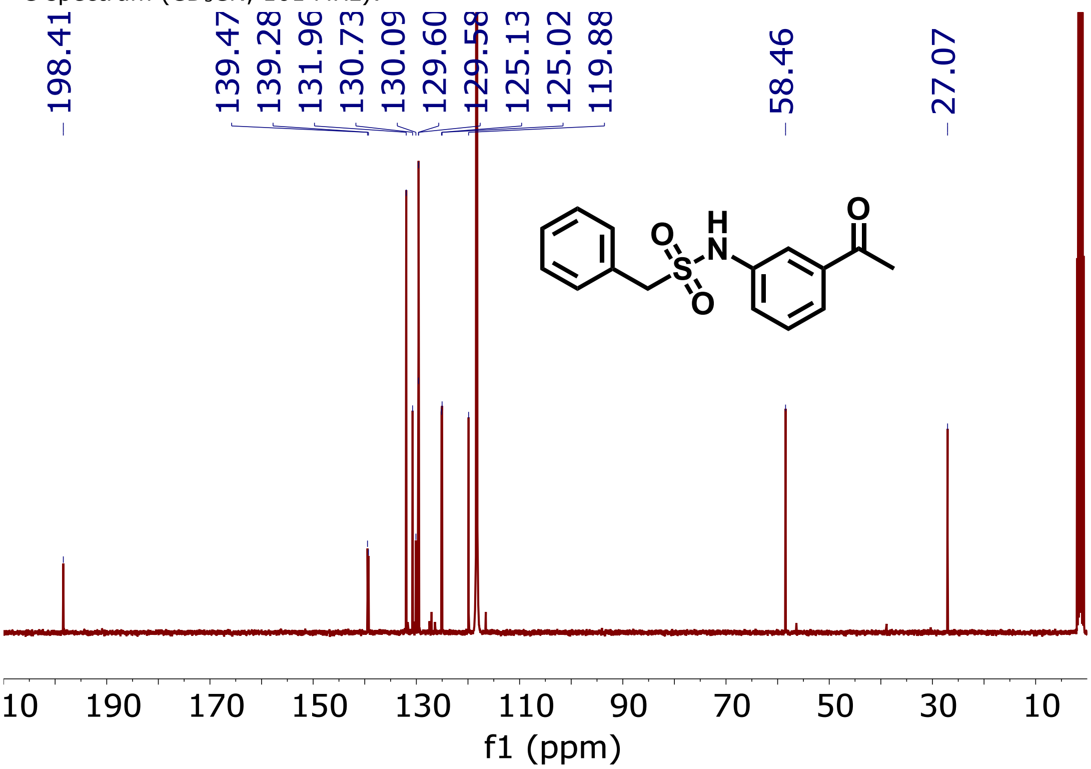

5q: *N*-(4-(4-aminophenoxy)phenyl)-1-phenylmethanesulfonamide

^{1}H spectrum (CD_3CN, 400 MHz):

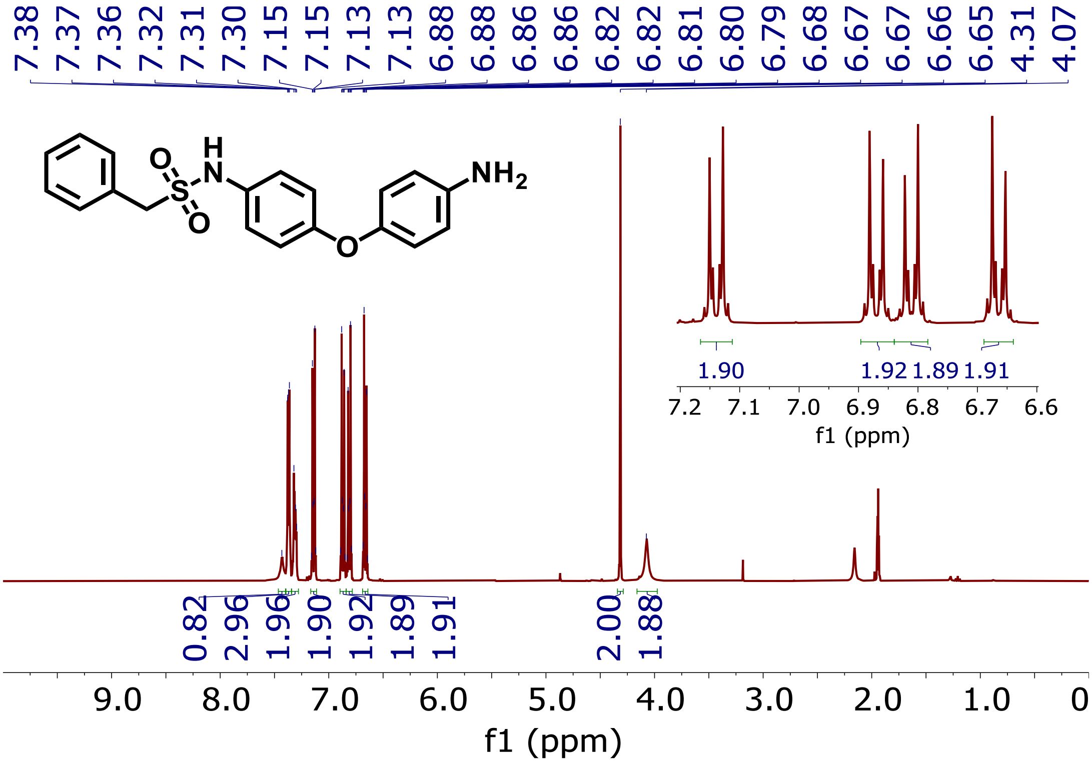

^{13}C spectrum (CD_3CN, 101 MHz):

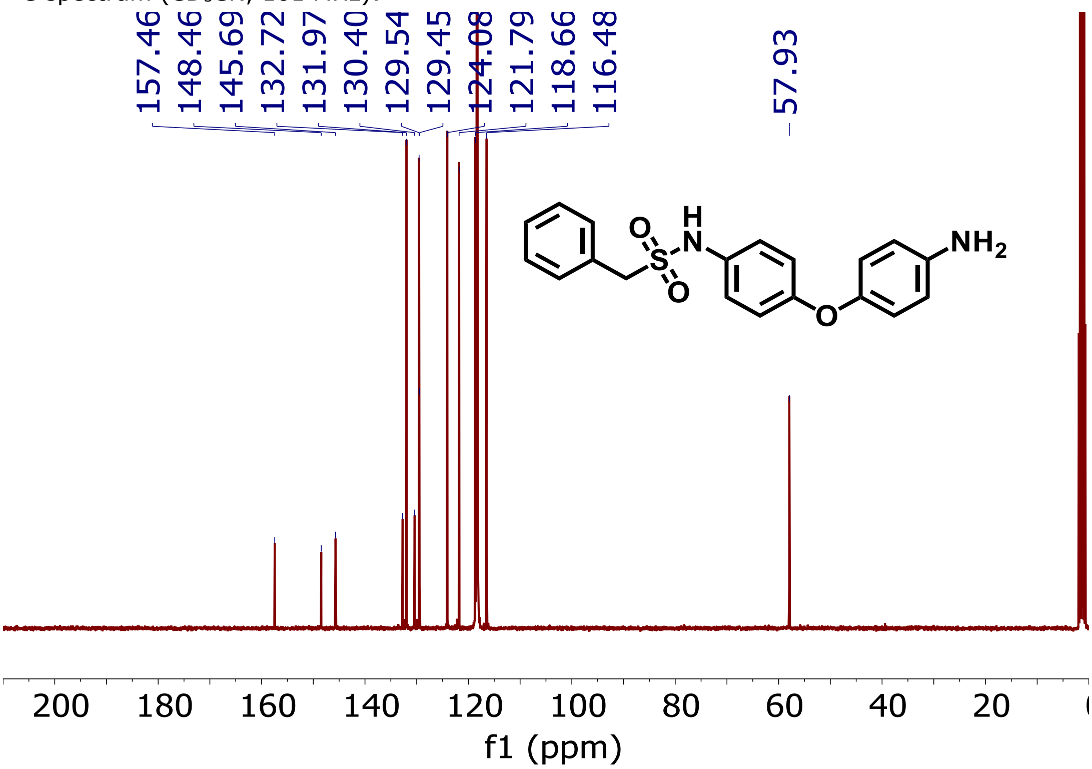

5s: 1-(benzylsulfonyl)-1*H*-indole

^{1}H spectrum ($CDCl_3$, 400 MHz):

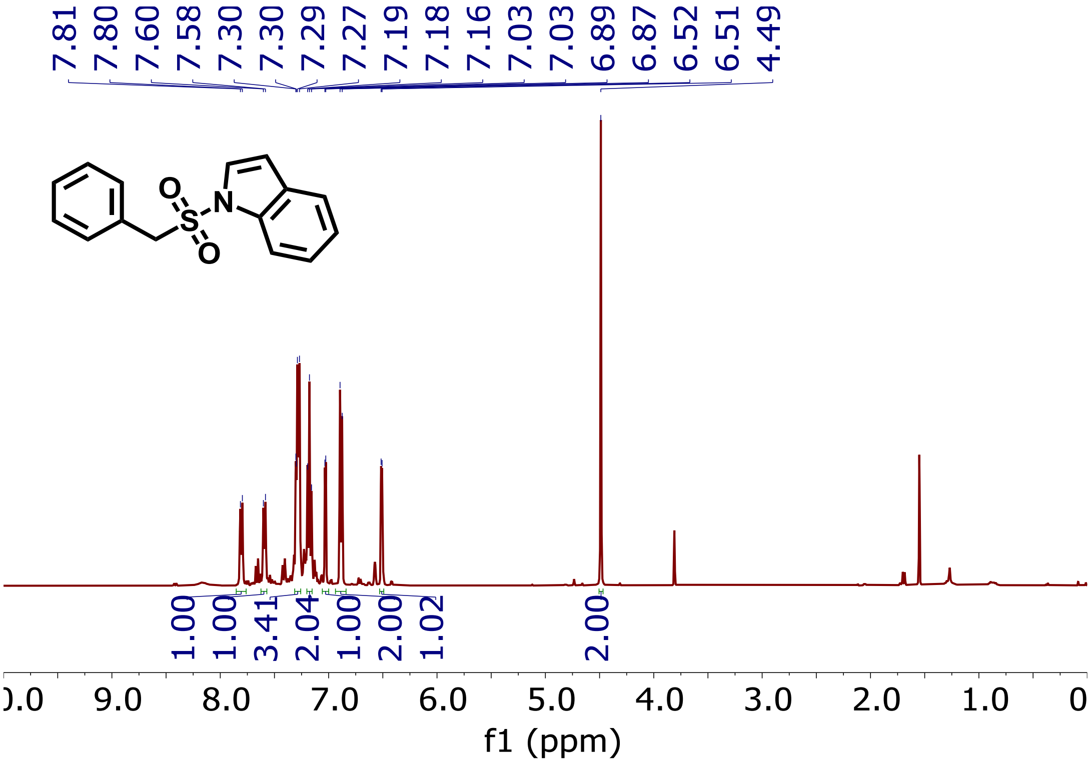

^{13}C spectrum ($CDCl_3$, 101 MHz):

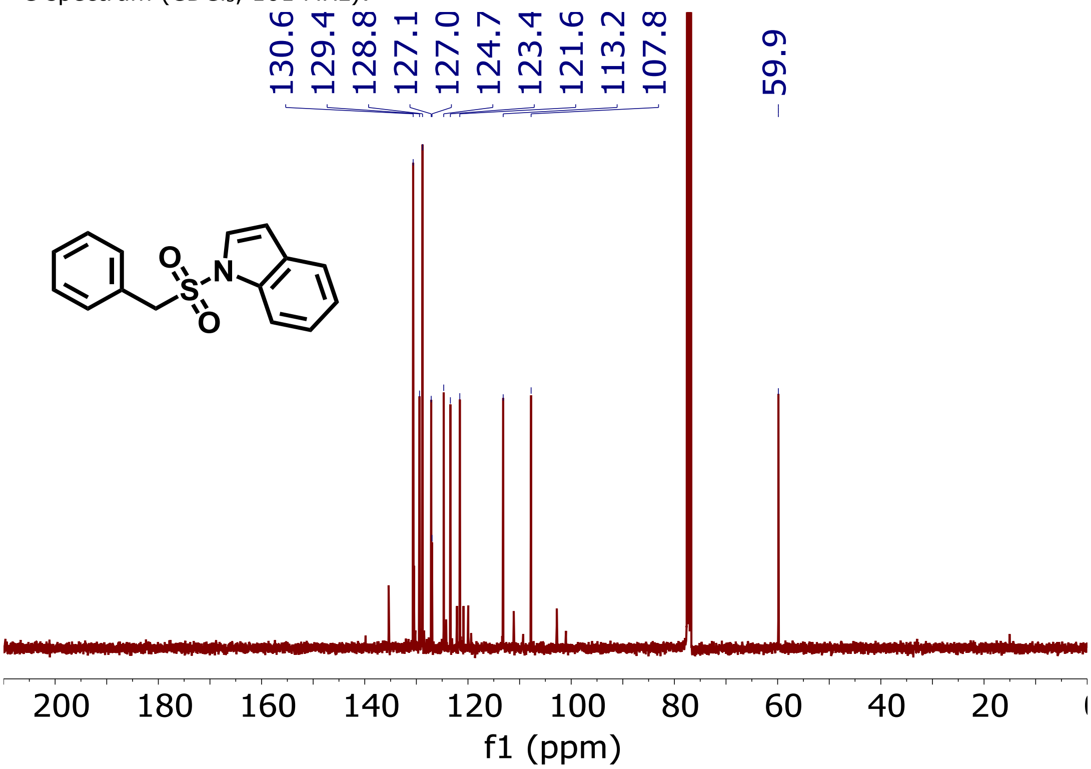

7c: 1-phenyl-*N*-(piperidin-1-yl)methanesulfonamide

^{1}H spectrum (CD_3CN, 400 MHz):

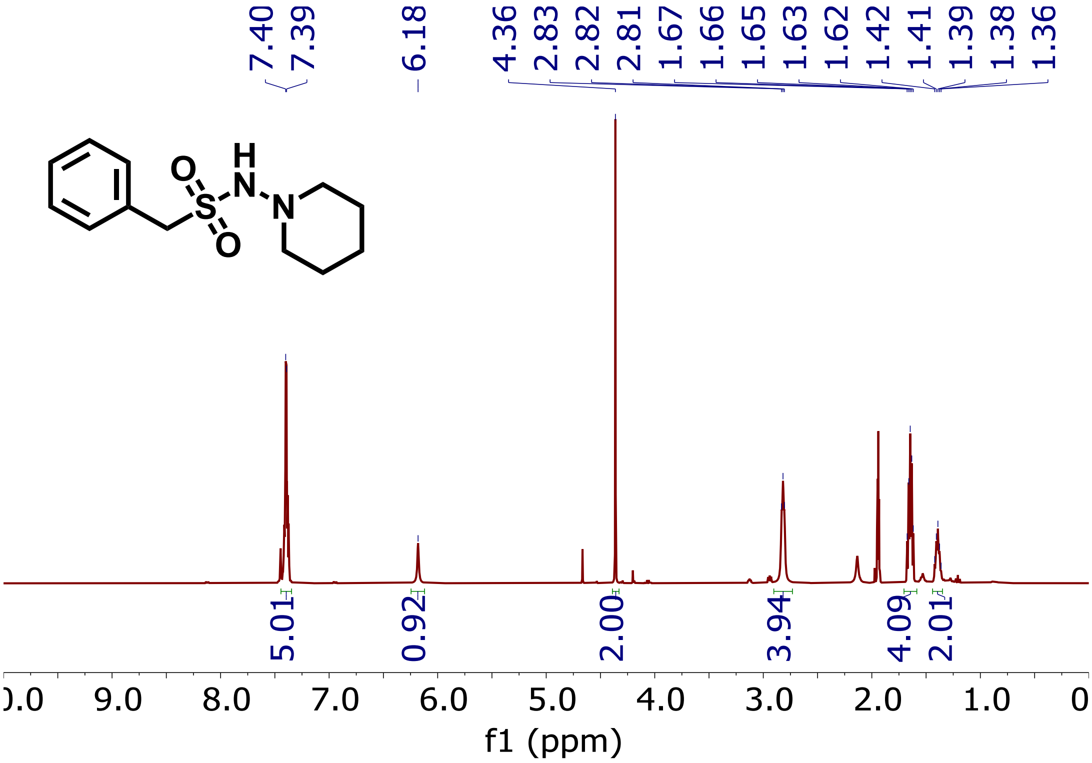

^{13}C spectrum (CD_3CN, 101 MHz):

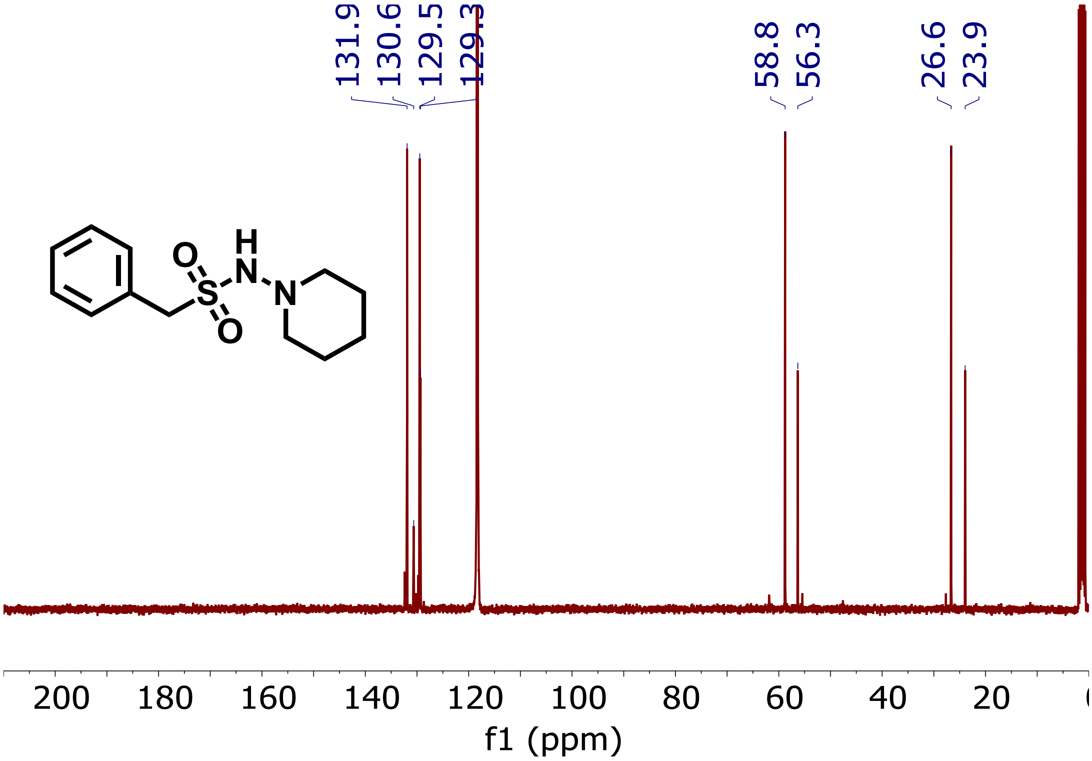

7d: *N*'-hexanoyl-1-phenylmethanesulfonohydrazide

^{1}H spectrum (CD_3CN, 400 MHz). A drop of ethyl acetate was added to prevent the compound from crystallizing out:

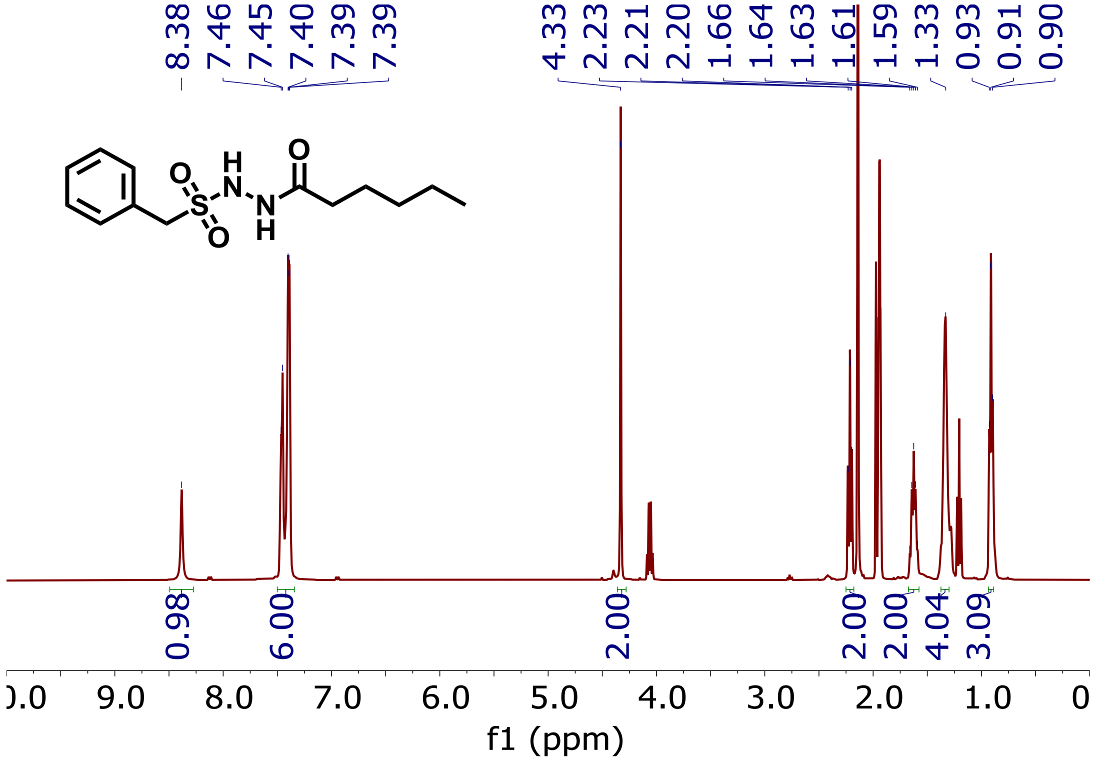

^{13}C spectrum (CD_3CN, 101 MHz). A drop of ethyl acetate was added to prevent the compound from crystallizing out:

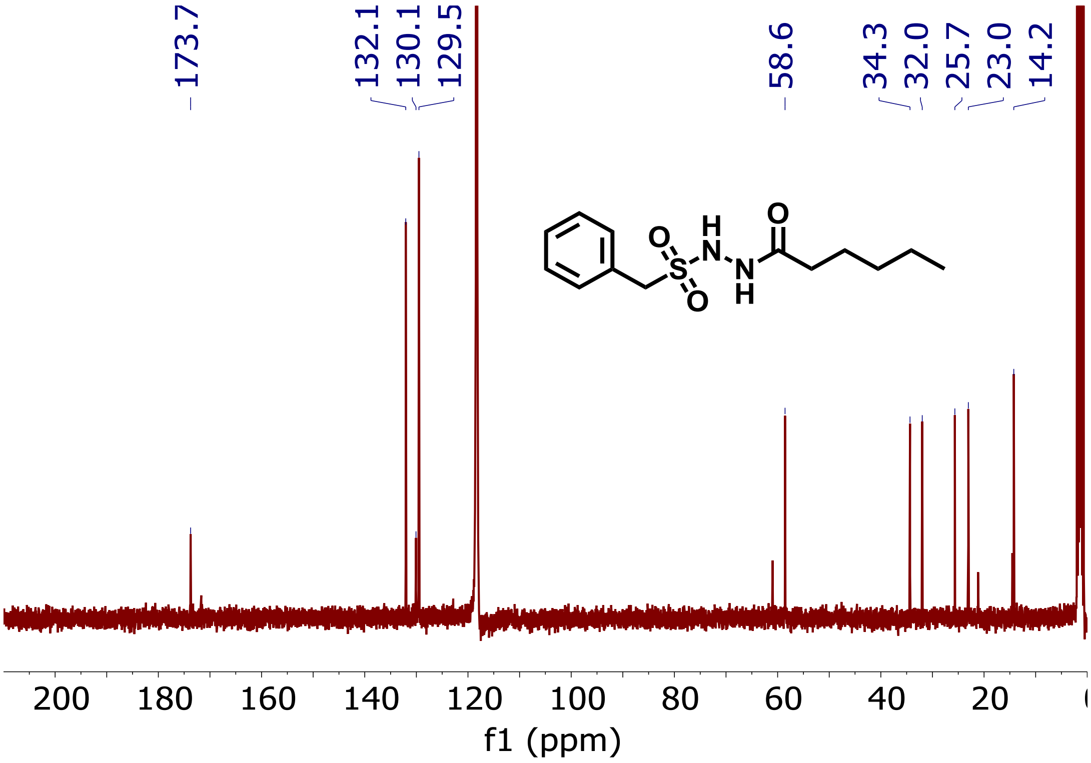

References

(1) van den Boom, A. F. J.; Subramaniam, M.; Zuilhof, H. Sulfur-Phenolate Exchange As a Fluorine-Free Approach to S(VI) Exchange Chemistry on Sulfonyl Moieties. (2022), *Org Lett*, **24** (47), 8621–8626.

(2) Day, J. J.; Neill, D. L.; Xu, S.; Xian, M. Benzothiazole Sulfinate: A Sulfinic Acid Transfer Reagent under Oxidation-Free Conditions. (2017), *Org Lett*, **19** (14), 3819–3822.

(3) Harger, M. J. P. Competing Sulfonylation and Phosphonylation Following Rearrangement of an O-Sulfonyl-N-Phosphinoylhydroxylamine with Tert-Butylamine: Demonstration of a Phosphonamidic-Sulfonic Anhydride Intermediate and 18O-Labelling Evidence on How It May Be Formed. (2003), *Org Biomol Chem*, **1** (19), 3390–3395.

(4) Johnson, T. B.; Ambler, J. A. Researches on Amines. IV. The Alkylation and Hydrolysis of Aliphatic Sulfonamides. A New Synthesis of Sarcosine. (1914), *J Am Chem Soc*, **36** (2), 372–385.

(5) Nikam, S. S.; Kornberg, B. E.; Ault-Justus, S. E.; Rafferty, M. F. Novel Quenchers for Solution Phase Parallel Synthesis. (1998), *Tetrahedron Lett*, **39** (10), 1121–1124.

(6) Zhou, G.; Ting, P.; Aslanian, R.; Piwinski, J. J. A Useful Pd-Catalyzed Negishi Coupling Approach to Benzylic Sulfonamide Derivatives. (2008), *Org Lett*, **10** (12), 2517–2520.

(7) Wojciechowski, K. Synthesis of Nitrobenzophenones from Nitro-α-Sulfonyldiphenylmethane Derivatives. (1997), *Synth Commun*, **27** (1), 135–144.

(8) Hill, B.; Liu, Y.; Taylor, S. D. Synthesis of α-Fluorosulfonamides by Electrophilic Fluorination. (2004), *Org Lett*, **6** (23), 4285–4288.

(9) King, J. F.; Kang, Y. I. Basic Catalysis in the Trapping of Sulphenes. (1975), *J Chem Soc Chem Commun*, No. 2, 52–53.

(10) Marvel, C. S.; Gillespie, H. B. Identification of Amines. III. Benzylsulfonamides. (1926), *J Am Chem Soc*, **48** (11), 2943–2944.

(11) Wydysh, E. A.; Medghalchi, S. M.; Vadlamudi, A.; Townsend, C. A. Design and Synthesis of Small Molecule Glycerol 3-Phosphate Acyltransferase Inhibitors. (2009), *J Med Chem*, **52** (10), 3317–3327.

(12) Abdulla, O.; Clayton, A. D.; Faulkner, R. A.; Gill, D. M.; Rice, C. R.; Walton, S. M.; Sweeney, J. B. Catalytic Sp3–Sp3 Functionalisation of Sulfonamides: Late-Stage Modification of Drug-Like Molecules. (2017), *Chemistry – A European Journal*, **23** (7), 1494–1497.
